# Structural biology of HIV-1 reverse transcriptase allosteric inhibitors for drug design

**DOI:** 10.1016/j.apsb.2025.11.007

**Published:** 2025-11-13

**Authors:** Zhenzhen Zhou, Yanying Sun, Da Feng, Zhao Wang, Fabao Zhao, Shenghua Gao, Peng Zhan, Dongwei Kang, Xinyong Liu

**Affiliations:** Department of Medicinal Chemistry, Key Laboratory of Chemical Biology (Ministry of Education), School of Pharmaceutical Sciences, Cheeloo College of Medicine, Shandong University, Jinan 250012, China

**Keywords:** HIV-1, Reverse transcriptase, Anti-HIV-1 drugs, NNRTIs, Structural biology, Allosteric inhibitors, Drug design, Drug resistance

## Abstract

HIV-1 reverse transcriptase (RT) is responsible for reverse transcription of viral single-stranded RNA to double-stranded DNA, which plays an important role in the replication cycle of HIV-1 and has been identified as a key target for anti-HIV-1 drug discovery. Among HIV-1 RT inhibitors, allosteric inhibitors acting on non-catalytic sites have the advantages of high efficiency and low cytotoxicity, which are the focus of the research on anti-HIV-1 inhibitors. Great progress has been achieved in the structural biology of HIV-1 RT, which significantly facilitated the development of RT allosteric inhibitors. Herein, we provided a detailed review of the co-crystal structures of small molecule allosteric inhibitors in complex with RT reported in the last decade. Moreover, the strategies to discover novel and efficient inhibitors based on co-crystal structures have also been discussed, expecting to provide a reference for the development of the next-generation anti-HIV-1 drugs.

## Graphical abstract

This is a detailed review of the co-crystal structures of small molecule allosteric inhibitors in complex with HIV-1 reverse transcriptase (RT) reported in the last decade.

**Figure:**
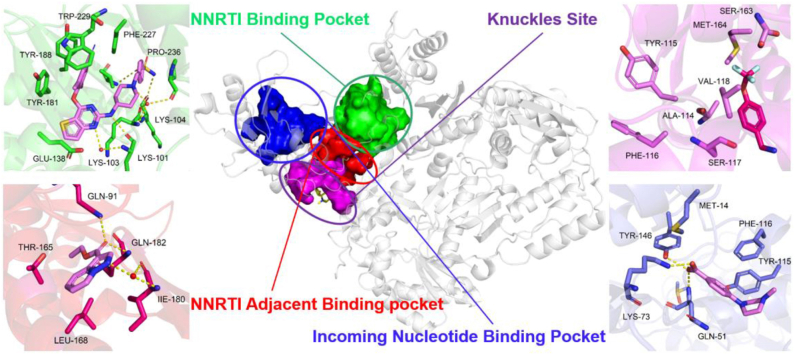

## 1 Introduction

Acquired immunodeficiency syndrome (AIDS), mainly caused by infection of the human immunodeficiency virus (HIV), was discovered in the 1980s with high infection rate and mortality. HIV can be divided into HIV-1 and HIV-2, among which HIV-1 is the main subtype responsible for the AIDS epidemic. HIV-1 can attack CD4^+^ T cells, triggering widespread immune abnormalities and increasing the risk of infection and tumor complication^1^. The advent of highly active antiretroviral therapy (HAART) has transformed ADIS from a highly lethal disease into a manageable chronic disease^2^. However, AIDS still is a major health problem with approximately 39.0 million people worldwide infected with HIV. In addition, 1.3 million new cases of infection were reported in 2022, and 630 thousand AIDS-related deaths every year^3^.

**Figure 1:**
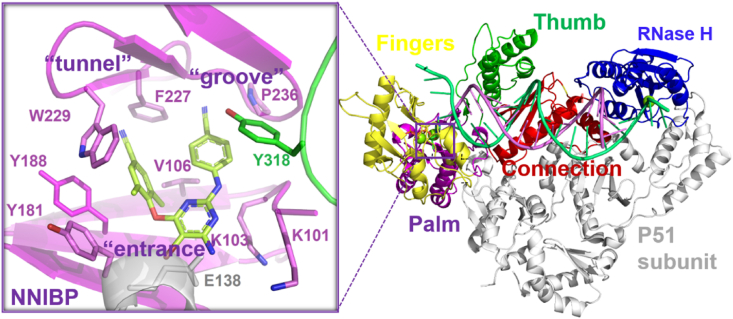
Overview of HIV-1 RT structure and NNIBP with inhibitor.

The key enzymes in the life cycle of HIV-1 virus have currently become the druggable anti-HIV targets, such as integrase, protease, and reverse transcriptase (RT)^4^, ^5^, ^6^, ^7^. Among them, reverse transcriptase (RT) plays an important role in the reverse transcription of single-stranded RNA into double-stranded DNA. RT is a heterodimer composed of p66 and p51 subunits, among which the p66 subunit consists of the N-terminal DNA polymerase domain and C-terminal RNase H domain, while p51 subunit is just a structural element without an active domain^8^. The shape of the polymerase domain is similar to that of a human right hand, so it is divided into four subdomains and named: fingers, palm, thumb, and connection, which can synthesize double-stranded DNA using viral single-stranded RNA as template (Fig. 1)^2^. The function of the RNase H domain is to catalyze the degradation of RNA/DNA hybrid strands, in preparation for DNA synthesis. Moreover, there is no homologous enzyme in the human body, making it an ideal drug target against HIV-1 virus.

**Figure 2:**
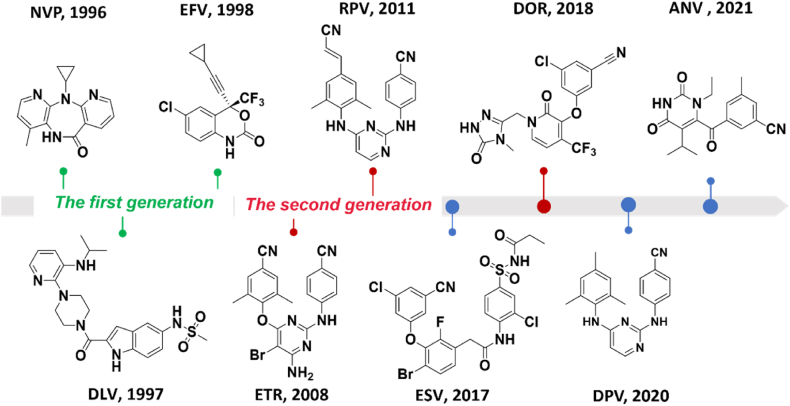
Structures of nine approved NNRTIs.

According to the mechanism of action, HIV-1 RT inhibitors can be divided into nucleos(t)ide reverse transcriptase inhibitors (N(t)RTIs) and non-nucleoside reverse transcriptase inhibitors (NNRTIs). Among them, NNRTIs bind to a hydrophobic pocket about 10 Å away from the polymerase active site, known as NNRTIs binding pocket (NNIBP) (Fig. 1). The pocket is allosteric, which does not exist in the natural HIV-1 RT, but it was allosterically formed once NNRTIs was combined^9^. Allostery is an effect that can change the functional activity of the protein by binding at a site distant from the active site. Allosteric is known as the “second secret of life” because of its direct and effective regulatory function, which plays a fundamental role in countless biological processes of all organisms. Drugs designed to target the allosteric sites can potentially achieve high selectivity and have some advantages (*e.g.*, long target action time). Since the first allosteric drug was approved by the US Food and Drug Administration (FDA) in 2004, it has been greatly developed in recent years^10^. Compared with normal ligands, allosteric modulators have the advantage of high selectivity for target proteins. Additionally, allosteric modulators do not compete with endogenous ligands *in vivo*, which allows them to be used in combination with the drugs acting on the active site and reduce side effects^11^. NNRTIs, as allosteric inhibitors, can alter the conformation of HIV-1 RT active site by binding to the allosteric site, thereby inhibiting the function of RT. Significantly, NNRTIs have been demonstrated to be a major component of HAART because of their potent activity, lower toxicity, and higher selectivity^12^, and their combinations with NRTIs are widely used in the first-line treatments for HIV/AIDS patients^13^. Until now, six NNRTIs have been approved by the FDA, including nevirapine (NVP), delavirdine (DLV), efavirenz (EFV), etravirine (ETR), rilpivirine (RPV) and doravirine (DOR). In addition, elsulfavirine (ESV), dapivirine (DPV), and ainuovirine (ANV) have also been approved by Russia, Europe, and China in 2017, 2020, and 2021, respectively (Fig. 2). However, the long-term clinical use of NNRTIs has resulted in serious viral resistance to them, greatly reducing their therapeutic effect on HIV-1/AIDS. Therefore, how to overcome the rapidly emerging drug resistance is an imminent and challenging need in the research community.

Structural biology is an interdisciplinary discipline that studies the three-dimensional spatial structure, dynamic process, and biological function of biological macromolecules, which plays a fundamental role in revealing the movement process of life and the pathogenesis of diseases. In drug discovery, structural biology reveals the binding mode of drugs and targets, which provides the basis for target-based precision drug design. As for HIV-1 RT, significant progress in structural biology has been achieved. In recent years, a large number of co-crystal structures of HIV-1 RT and NNRTIs have been reported to illustrate their unique binding modes. In this work, we provide a detailed review of the advances in co-crystal structures of HIV-1 allosteric inhibitors, with the hope to provide valuable insights for combating the drug resistance and the discovery of next-generation RT inhibitors.

## 2 The classical allosteric site of HIV-1 RT: NNIBP

**Figure 3:**
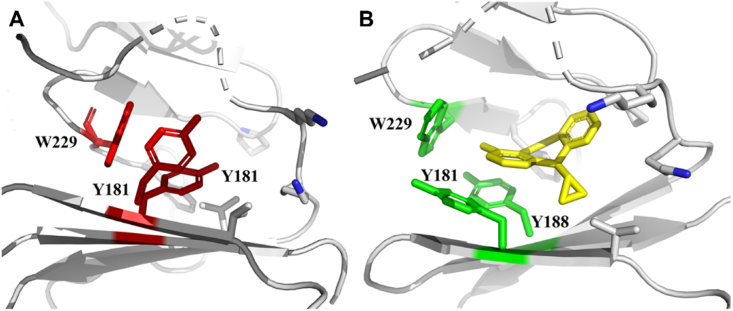
(A) Crystal structure of key amino acids prior to NNRTIs binding (PDB code: 1T03). (B) Crystal structure of the NNIBP of HIV-1 RT (PDB code: 1VRT).

The crystallographic studies demonstrated that NNIBP is absent from RT in the absence of NNRTIs binding (Fig. 3A)^14^. Actually, Y181 and Y188 rotate to the active site to cause a conformational change when NNRTIs bind to RT^15^, forming NNIBP to accommodate NNRTIs (Fig. 3B)^16^, ^17^, ^18^. NNIBP mainly consists of K101, K103, V106, Y181, Y188, F227, W229, P236, Y318 of p66 and E138 of p51 (Fig. 1). Bec et al.^19^ found that the inhibition of NNRTIs is primarily due to the presence of NNIBP, which prevents RT from correctly binding DNA in a catalytic manner, leading to the formation of terminal RT/DNA/NNRTI complexes that cannot bind afferents the incoming nucleotide substrate.

Since the residues of NNIBP are not directly involved in the polymerization process of RT, the NNIBP residues have little cost of mutation, while the potency of NNRTIs is reduced or even lost^20^. The NNIBP mutations could be divided into the following three categories: the loss of hydrophobic interactions, mutation of residues at the entrance channel of the NNIBP, and changes in central steric hindrance. The key amino acid residues located in the hydrophobic interaction region of NNIBP are Y181, Y188, and F227^21^, the mutations (Y181C, Y188 L/C, and F227C) result in a loss of hydrophobic interactions between NNRTIs and NNIBP, which result in the reduced activity of NNRTIs. The mutations at the entrance channel of NNIBP refer to K101 E/P, K103N, and E138K^22^, their side chains can stick out of the pocket and prevent the entry of NNRTIs^23^. And, mutations in L100I and G190A cause a change in the central steric hindrance of NNIBP, changing the conformation of the pocket. In conclusion, mutations of the key amino acid residues in NNIBP cause severe drug resistance. Therefore, this chapter analyzed the binding modes of the FDA-approved NNRTIs with wild-type (WT) and mutant HIV-1 RT and summarized the advances in the co-crystal structures of NNRTIs with HIV-1 RT to reveal how to address drug resistance from the perspective of drug–target interaction and to assist the launch of the nest generation NNRTIs.

### 2.1 The structural biology studies of approved NNRTIs with HIV-1 RT

#### 2.1.1 The first-generation NNRTIs

**Figure 4:**
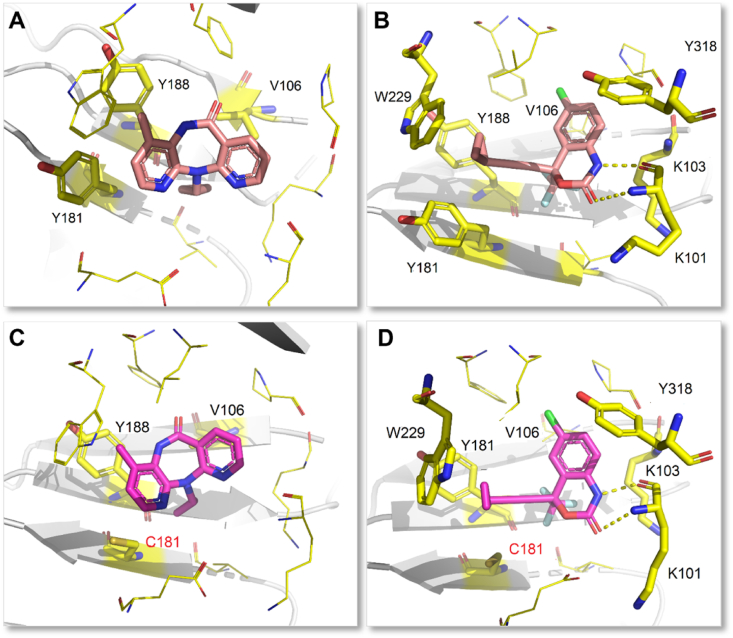
(A) Crystal structure of HIV-1 WT RT in complex with NVP (PDB code: 7KJX). (B) Crystal structure of HIV-1 WT RT in complex with EFV (PDB code: 1IKW). (C) Crystal structure of HIV-1 Y181C RT in complex with NVP (PDB code: 1JLB). (D) Crystal structure of HIV-1 Y181C RT in complex with EFV (PDB code: 1JKH). Hydrogen bonds are shown as yellow dotted lines.

Fig. 4A shows the conformation of NVP in NNIBP. As previously mentioned, the residues Y181 and Y188 rotate toward the active site to accommodate NVP^16^. Remarkably, the interactions that maintain the conformational stability of the complex are the hydrophobic and stacking interactions formed by the two pyrimidine rings, as well as the alkyl substitutions of NVP with V106 and the aromatic side-chain substitution of Y188 and Y181. In contrast to NVP, EFV develops a double-hydrogen bonds between the benzoxazin-2-one NH and carbonyl group with the backbone of K101. Besides, the benzoxazin-2-one ring is close to V106, making contact with Y318, and since the *N*-atom on the ring is also located near K103, it allows van der Waals interactions (Fig. 4B)^24^.

**Figure 5:**
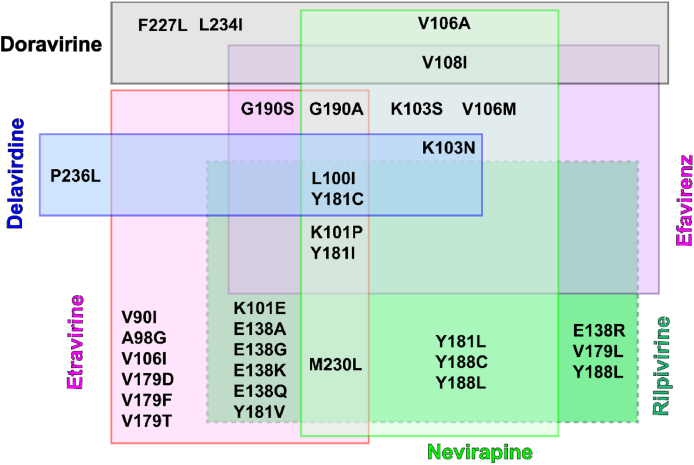
The main resistant mutants of FDA-approved NNRTIs.

The resistance of NVP is mainly caused by the mutations of one or several amino acid residues in K101I, G190A, K103 N/S, V106 A/M, V108I, Y181 C/L, Y188 C/L, and M230L^25^. Among them, Y181C, Y188L, and K103N are the most common mutant strains in clinical, which results in a significant reduction in NVP efficacy. The Y181/Y188 residue mutated to C181/C188 caused the disappearance of the aromatic ring stacking interactions between NVP and NNIBP, which accounts for the reduced antiviral activity (Fig. 4C)^24^. Besides, the mutations G190A and L100I affected the flexibility and conformation of their surrounding residues, which sterically hindered the binding of the rigid molecule NVP to NNIBP^26^. EFV has approximately the same resistance mutants as NVP. However, EFV has a better barrier to resistance compared to NVP (Table 1). One important reason is that the aromatic ring stacking interactions of EFV with Y181 and Y188 are weaker than that of NVP, which is due to the smaller propynyl-cyclopropyl group of EFV, as reflected by the co-crystal structure of EFV with Y181C RT (Fig. 4D)^27^. Compared with NVP, EFV was less sensitive to K103N mutant strain^28^. The rearrangement ability of EFV is stronger than that of the rigid molecule NVP in the mutant K103N NNIBP, which may explain the greater resistance of EFV^27^. The inhibition of EFV by G190A and KL00I is consistent with that of NVP. In addition, mutant strains of EFV also include K101P, K103S, V106M, V108I, and G190 A/S (Fig. 5), but co-crystal structures of EFV and these mutant strains have not been reported^25^.

**Table 1:** Activity against mutant HIV-1 strains of NVP, EFV, ETR, and RPV^29^.

NNRTI	EC_50_ (nmol/L)							
	WT	L100I	K103N	Y181C	Y188L	E138K	F227L/V106A	K103N/Y181C
NVP	85	600	>1000	>1000	>10,000	ND	ND	>10,000
EFV	3.0	115	80.1	8.2	514	5.2	348.5	NA
ETR	3.5	11.7	3.7	16.6	15.4	18.8	26.9	52.2
RPV	1.0	1.5	1.3	4.73	79.4	5.75	81.6	10.7

NA for EC_50_ > CC_50_; ND for not determined.

#### 2.1.2 The second-generation NNRTIs

**Figure 6:**
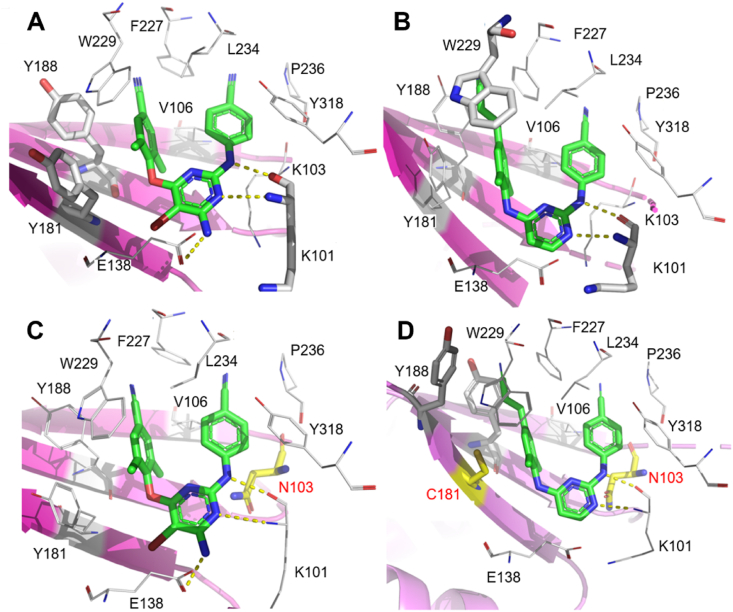
(A) Crystal structure of HIV-1 RT in complex with ETR (PDB code: 3MEC). (B) Crystal structure of HIV-1 RT in complex with RPV (PDB code: 2ZD1). (C) Crystal structure of K103N mutant HIV-1 RT in complex with ETR (PDB code: 3MED). (D) Crystal structure of K103N/Y181C mutant HIV-1 RT in complex with RPV (PDB code: 3BGR).

Etravirine (ETR) and Rilpivirine (RPV) are known as the second-generation NNRTIs, both of them belong to the diarylpyrimidine (DAPY) family and adopt a horseshoe-like conformation in NNIBP, exhibiting much improved activity against mutant strains compared to that of the first-generation NNRTIs^29^. The DAPY NNRTIs adopted a typical three-point pharmacophore model, including hydrophobic interaction region, hydrogen binding domain, and tolerant region I. The left wing of ETR is located in the hydrophobic interaction region composed of aromatic amino residues Y181, Y188, F227, and W229, and forms *π‒π* interactions with these residues, which are considered to be the dominant force for the combination of inhibitors and NNIBP. Double hydrogen bonds can be observed in the hydrogen binding domain, the N atom of the pyrimidine ring and the NH linker between the central pyridine ring and the right ring develop the “signature” hydrogen bond with the backbone of K101 (Fig. 6A), which are not lost by mutations of the amino acid residues^30^. Besides, the primary amine group can form a hydrogen bind with the carbonyl group on the E138 side chain. The benzonitrile extends into tolerant region I consisting of V106, L234, P236, and Y318, forming extensive van der Waals interaction with these residues. As for RPV, it adopts the same conformation in NNIBP with that of ETR (Fig. 6B). Although RPV lost the hydrogen bond with E138, the cyano vinyl group of RPV could forward to reach the hydrophobic tunnel formed by F227 and W229 and develop additional interactions with the highly conserved W229.

Although numbers of resistance-associated mutations (RAMs) to ETR and RPV have been reported (Fig. 5), both of them showed improved drug resistance profiles against the dominant single mutant strains (L100I, K103N, and Y188L) compared to the first-generation NNRTIs^31^^,^^32^. Due to the highly flexible conformation, ETR and RPV can adapt to the structural changes by torsional flexibility (“wiggling”), repositioning, and reorientation (“jiggling”) (Fig. 6C). However, the double and triple mutants also led to a significantly decreased activity of ETR, such as a combination of L100I, K103N, Y181C, G190E, M230L, and Y318F. For example, the most common double NNRTIs-resistant strain K103N/Y181C resulted in reduced potency of ETR (EC_50_ = 52.2 nmol/L), but RPV exhibited an EC_50_ value of 10.7 nmol/L and was more potent than that of ETR (Table 1)^29^. The co-crystal structure of RPV in complex with K103N/Y181C RT demonstrated that the interaction of the cyanovinyl group of RPV with Y183 could compensate for the loss of *π‒π* interactions caused by the Y181 mutation (Fig. 6D)^31^. Meanwhile, the extent of interactions between cyanovinyl and the hydrophobic tunnel is conserved, which leads to significantly improved drug resistance profiles than ETR. Although RPV can adapt to some mutants with its flexible structure, decreased susceptibility to RPV has been observed in K101 E/P, E138 A/G/K/Q/R, V179L, Y181I/V, H221Y, F227C, and M230I/L mutant strains^33^. It has been reported that the K101P mutation may cause misincorporation of RT, making it more susceptible to other mutations and making it 50-fold less sensitive to NVP^34^^,^^35^. The co-crystal structure of the NNRTIs **25a**/K101P RT was determined in our previous work, the superposition results of co-crystal structure showed that K101P mutation can eliminate the hydrophobic interaction between the pyrimidine ring of RPV and the long aliphatic side chain of K101. Moreover, it may introduce the bad spatial collision between pyrimidine ring and the nonpolar side chain of P101^36^. The combination of E138 A/G/K/Q/R and M184I mutants can also significantly reduce the potency of RPV^37^. Recently, it has been reported that DNA localization relative to I184 and opening of the NNIBP entrance by the E138K mutation could contribute to the drug resistance^38^. For example, the E138K mutation disrupted the hydrophobic interactions between the RPV central pyrimidine and the residue V179, increasing the rate of RPV dissociation from NNIBP^36^. When M184I and E138K are combined, the antiviral activity to RPV is reduced approximately 7-fold, whereas M184I alone does not reduce the susceptibility of RPV^39^.

#### 2.1.3 Doravirine

**Figure 7:**
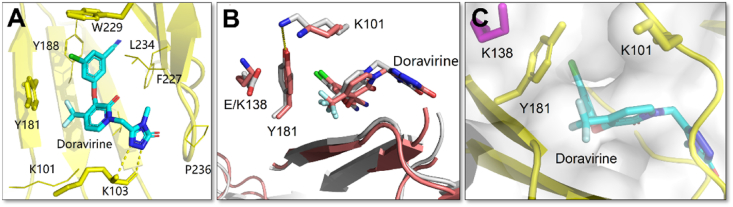
(A) Crystal structure of HIV-1 RT in complex with DOR (PDB code: 4NCG). Select residues of the NNIBP of HIV-1 RT are shown as pink sticks, with DOR shown as yellow sticks. Hydrogen bonds are shown as yellow dotted lines. (B) Superposition of WT RT/DOR complex structure (grey) onto E138K/M184I RT/DOR complex structure (orange). (C) Crystal structure of HIV-1 E138K/M184I RT in complex with DOR (PDB code: 7Z2H).

Doravirine (DOR) is the latest FDA-approved (August 2018) NNRTIs for the treatment of HIV-1-infected adults. As depicted in Fig. 7A, the conformation of DOR in the NNIBP also resembles the “horseshoe shape”^40^. The left hydrophobic benzene substituent forms *π‒π* stacking interactions with Y188 and the conserved residue W229, the triazole extends into a sub-pocket surrounded by L234, F227, V106, and P236 and forms extensive van der Waals interactions with these residues. Different from the second-generation NNRTIs, the triazole of DOR develops double hydrogen bonds with the backbone of K103, which plays a vital role in its binding mode with NNIBP^41^.

DOR maintains high activity against the single mutant strains (Y181C, K103N, and G190A) and the double mutant strains (K103N/Y181C and E138K/M184I), which are most likely to appear in the clinical use of NNRTIs^42^. Specifically, DOR was 4-10-fold more active against Y181C mutant and 7-12-fold more active against K103N/Y181C mutant compared to that of ETR and RPV, respectively (Table 2)^43^. As shown in Fig. 7A**,** DOR discarded the *π‒π* interactions with Y181 and interacted with the more conserved residue W299, which may explain the improved potency of DOR against Y181C. It is reported that the hydrogen bound formed between the Y181 side chain and the K101 side chain at the NNIBP entrance prevents the dissociation of NNRTIs and NNIBP and is essential for maintaining inhibitory activity (Fig. 7B). The loss of this hydrogen bond in E138K/M184I RT greatly reduced the susceptibility of ETR and RPV to E138K/M184I mutant. Although this hydrogen bond also disappeared in the E138K/M184I RT/DOR co-crystal structure, the side chain of Y181 still blocked the NNIBP entrance, which could prevent faster dissociation of DOR from NNIBP, leading to the high susceptibility of DOR to E138K/M184I mutant strain (Fig. 7C). Even so, decreased DOR activity has been reported to be associated with the single V106, F227 and L234I mutant, resulting in a more than 10-fold reduction in susceptibility of DOR^44^. Importantly, the triple mutants V106A/L234I/F227L and V108I/L234I/V106A could reduce DOR susceptibility by more than 150-fold, but these mutant strains are uncommon in clinic^42^. In addition, mutant strains of DOR also include V106I/M/T, Y188 L/H, G190E, P225H, F227 L/R, and M230L (Fig. 5), the co-crystal structures of DORV and these mutant strains have not been reported^25^.

**Table 2:** Antiviral activity of DOR, EFV, and ETR.

NNRTI	EC_95_ (nmol/L)			
	WT	K103N	Y188C	K103N/Y188C
ETR	20	42	27	55
RPV	38	36	263	653
DOR	37	48	120	407

#### 2.1.4 Elsulfavirine

**Figure 8:**
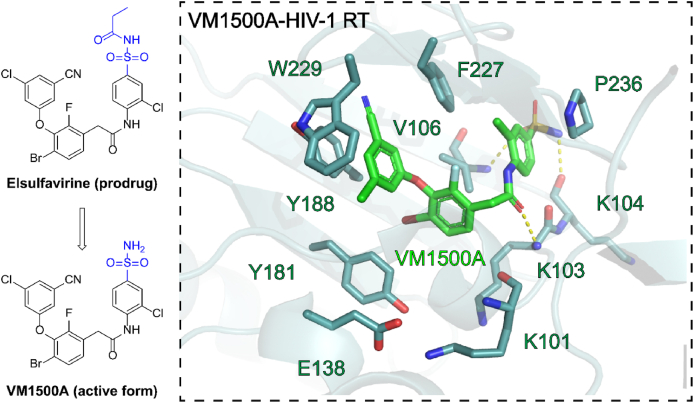
(A) Crystal structure of HIV-1 RT in complex with ESV (PDB code: 7TAZ).

Elsulfavirine (ESV) is a propanoyl sulphonamide prodrug form of its active form, the new-generation NNRTI, **VM1500A** (Fig. 8). As a drug candidate, ESV was discovered by Roche and received its first global approval on 30 June 2017, in Russia, for the treatment of HIV-1 infectious in combination with other antiretroviral drugs^45^. Although the resistance profile of ESV has not been reported yet, the co-crystal structure of its active form **VM1500A** and HIV-1 RT has been elucidated in 2023. As shown in Fig. 8, **VM1500A** adopts a binding mode similar to DOR, with an overall higher configuration in NNIBP. The specific effects of this form are: (1) the left ring abandoned the interaction with the mutable residue Y181, thereby strengthening the *π‒π* interaction with the highly conserved residue W229; (2) the amide linker lost the classic double hydrogen bond with K101 and only formed a hydrogen bond with K103; (3) its sulfonamide group extended into the solvent-exposed surface and formed extensive hydrogen bonds with the surrounding residues V106 and K104.

### 2.2 Advances in structural biology of NNRTIs

#### 2.2.1 Diarylpyrimidines (DAPYs)

DAPYs are considered a representative class of promising NNRTIs with potent anti-HIV-1 activity and have contributed to the discovery of two FDA-approved drugs ETR and RPV. However, drug resistance, poor pharmacokinetic profiles, and some adverse effects have been reported. Extensive efforts have been made for the structural modification of DAPYs to discover more potent HIV-1 NNRTIs with improved pharmacokinetic and safety profiles. Here, the co-crystal structures of different structural types of compounds with RT are summarized, and the binding mode between NNRTIs and RT is also analyzed, with the hope to shed light on the subsequent structural modification of DAPYs derivatives.

##### 2.2.1.1 K-5a2, 25a, 24b and 16c

**Figure 9:**
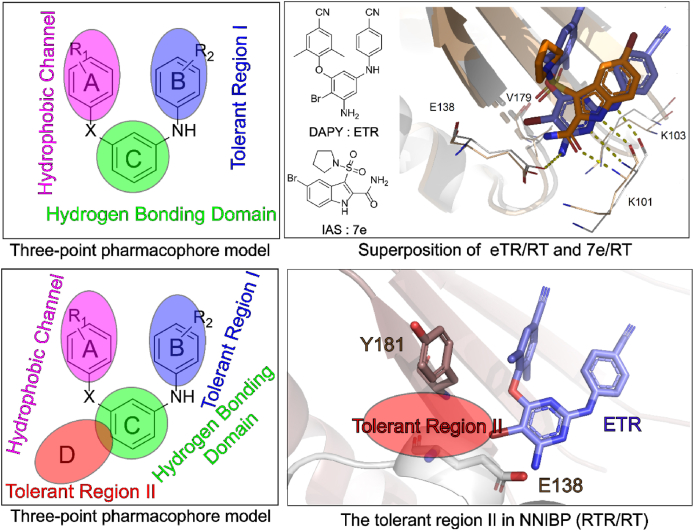
The proposal of DAPYs four-point pharmacophore model.

Based on the superposition of co-crystal structures ETR/RT (DAPYs) and **7e**/RT (IAS) and the three-point pharmacophore model of DAPYs, we first proposed a four-point pharmacophore model that can be targeted to tolerant region II of NNIBP, with the aim to improve the drug resistance profiles through a multi-site binding strategy (Fig. 9). This region is located at the junction of p66 subunit and p51 subunit interface at the bottom of NNIBP, which is another protein solvent interface with large modification space.

**Figure 10:**
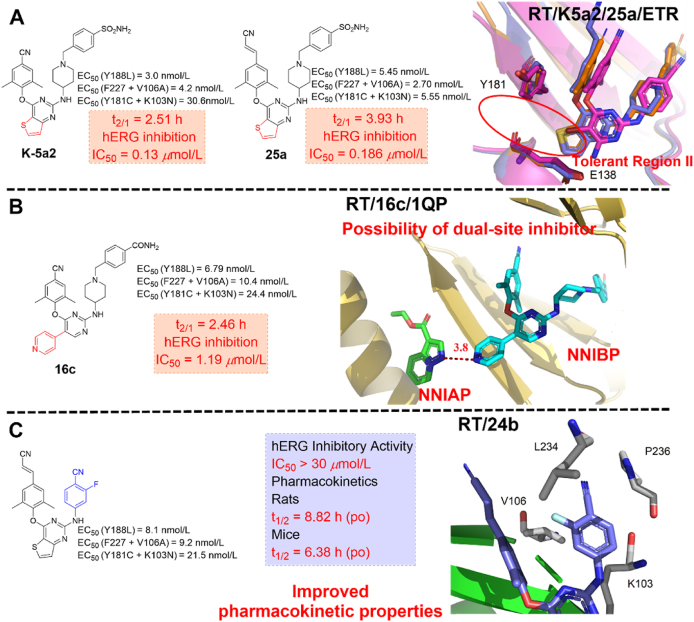
Our work based on the four-point pharmacophore model.

According to the newly proposed model, a series of novel DAPYs NNRTIs were designed in our lab and identified compounds with significantly improved drug resistance profiles (Fig. 10). Specifically, the anti-HIV-1 activity of **K-5a2** and **25a** against the common NNRTIs-resistant strains increased by 3–10 times compared with that of ETR^32^^,^^46^. The co-crystal structures of HIV-1 WT RT and seven RT variants bearing prevalent drug-resistant mutations in complex with **K-5a2** and **25a** have been determined to clarify their detailed interactions with RT. Encouragingly, the structure validates our proposed four-point pharmacophore model. Moreover, the molecular details of the extensive hydrophobic interactions and the network of backbone hydrogen bonds developed between the NNRTIs and NNIBP account for their potent activity against NNRTIs-resistant mutations^36^.

**Figure 11:**
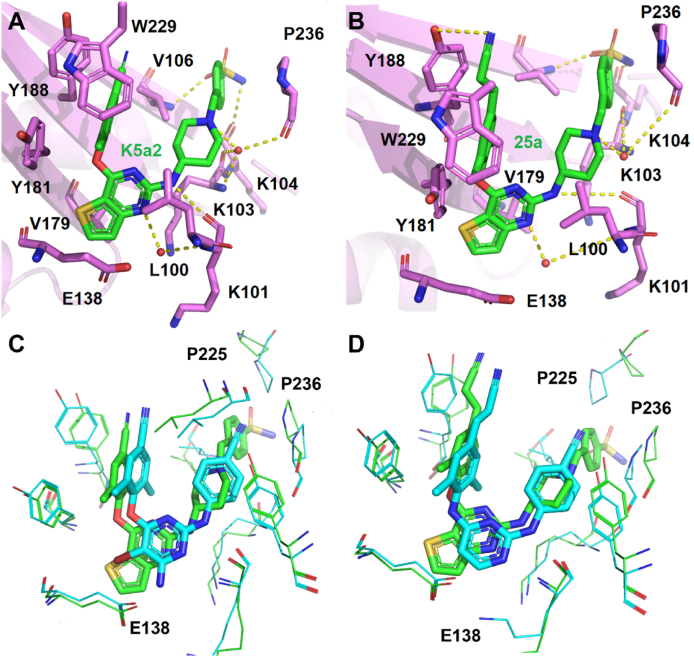
(A) Crystal structure of HIV-1 WT RT in complex with **K-5a2** (PDB code: 6C0J). (B) Crystal structure of HIV-1 WT RT in complex with **25a** (PDB code: 6C0N). Select residues of RT are shown as purple sticks, with **K-5a2 or 25**a shown as green sticks. (C) Superposition of WT RT/ETR complex structure (blue) onto WT RT/**K-5a2** complex structure (green). (D) Superposition of WT RT/RPV complex structure (blue) onto WT RT/**25a** complex (green) structure. Water molecules are shown as red spheres.

Similar to the second-generation NNRTIs ETR and RPV, **K-5a2** and **25a** adopted the classical horseshoe-like conformation in NNIBP, and their structures extend greatly into the three channels (entrance, tunnel, and groove) and show significant structural complementarity to NNIBP. As shown in Fig. 11A and B, the left wing of **K-5a2** or **25a** is located in the hydrophobic tunnel, and forms *π‒π* interactions with Y181, Y188, and W229. Especially, the cyanovinyl of **25a** can form further hydrophobic interactions with the highly conserved residue W229. In addition, the same central thiophene[3,2-*d*]pyrimidine core maintains favorable hydrophobic contacts with L100 and V179 but develops additional non-polar interactions with the alkyl chain of E138 compared to that of ETR and RPV. Their right-wing extended into the tolerant region I and formed extensive van der Waals contacts with K103, V106, and P236, and the terminal sulfonamide group was located on the solvent-exposed surface of RT^36^. In particular, multiple hydrogen bonds between **K-5a2** or **25a** and NNIBP were observed, including (1) a water-mediated hydrogen bond between the N atom in thiophene[3,2-*d*]pyrimidine ring and the NH on the K101 backbone; (2) a hydrogen bond formed by NH linker connecting the central thiophene[3,2-*d*]pyrimidine and right piperidine wing with the carbonyl of K101; (3) multiple network hydrogen bonds between the piperidine N atom with the main chain of K103 and P236 through a same water atom; and (4) two hydrogen bonds formed by the sulfonamide group with the main chain of V106 and K104. Moreover, the left-wing cyanovinyl group of **25a** formed an additional hydrogen bond with Y188 compared to that of **K-5a2**. In summary, the central favorable thiophene[3,2-*d*]pyrimidine core and the extensive hydrogen bonds interactions with the backbone of residues are less susceptible to amino acid mutations and could lock the inhibitors firmly in the pocket to inhibit polymerase activity, leading to excellent anti-HIV-1 activity of **K-5a2** and **25a** against the NNRTIs-resistant strains (Fig. 11C and D).

Meanwhile, further structural modifications have also been conducted to exploit the tolerant region II^47^, the target compound **16c** yielded the most active potency and have nanomolar potency against all tested strains, with EC_50_ values ranging from 3.75 nmol/L (WT), 4.26 nmol/L (L100I), 3.79 nmol/L (K103N), 6.79 nmol/L (Y181C), 6.79 nmol/L (Y188L), 10.9 nmol/L (E138K), 10.4 nmol/L (F227L/V106A) and 24.4 nmol/L (K103N/Y181C). Specifically, the potency of **16c** against single mutant strain Y188L and double mutant strain F227L/V106A was much increased, being about 11- and 7.8-fold more potent than that of **K-5a2**, respectively. Importantly, the discovery of **16c** provided a possibility for the design of RT dual-site inhibitors (Fig. 10B).

**Figure 12:**
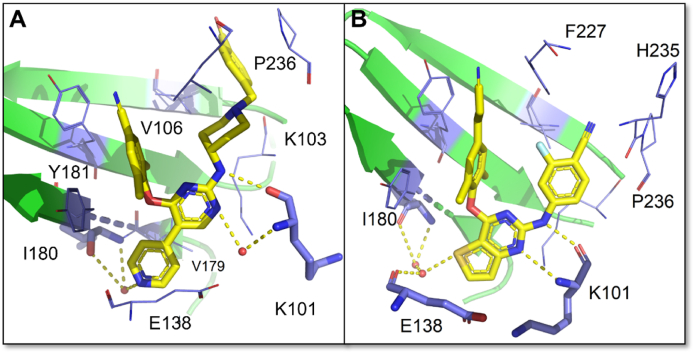
(A) Crystal structure of HIV-1 WT RT in complex with **16c** (PDB code: 7KWU). (B) Crystal structure of HIV-1 WT RT in complex with **24b** (PDB code: 6UL5).

Compared with **K-5a2**, the replacement of sulfonamide with an amide group results in the disappearance of the hydrogen bonds between the **16c** and V106, and K104 (Fig. 12A)^44^^,^^47^. However, the newly introduced 4-pyridyl substituent extends to the tolerant region II, forming more effective hydrophobic interactions with V179 and Y181 and additional double water-mediated hydrogen bonds with I180, which contribute to its potent activity. More importantly, the co-crystal structure revealed the feasibility of designing novel “dual-site”-binding allosteric inhibitors targeting both NNIBP and the NNRTIs adjacent site of RT (see Section 3.1 for details).

However, the easily metabolized and hERG-perturbing piperidine-linked benzene scaffold resulted in a shorter half-life (*t*_1/2_ = 2.51‒3.93 h) and higher hERG inhibition (0.13‒1.19 μmol/L) of **K-5a2, 25a** and **16c**. Based on the co-crystal structures **25a**/RT, a series of fluorine-substituted diarylpyrimidine derivatives were designed to improve the pharmacokinetic properties^48^. As excepted, compound **24b** not only exhibited excellent activity (EC_50_ = 3.60‒21.5 nmol/L) against NNRTIs-resistant strains but also had much reduced hERG inhibition (IC_50_ > 30 μmol/L) and higher half-life (*t*_1/2_ = 8.82 h) in rats (Fig. 10C).

Compared with **25a**, the S atom on the central thiophene ring of **24b** forms an additional water-mediated multiple hydrogen bonds network with the main-chain atoms of E138 and I180 (Fig. 12B). Meanwhile, the water between **24b** and E138 is conserved in the complexes of HIV-1 RT with RPV and **24b**. Interestingly, the CN substituent of the right wing interacts well with the carbonyl of H235 and the complementary electrostatic alignment of the two groups contributes to the remarkable potency of **24b** against drug-resistant HIV-1 strains.

##### 2.2.1.2 Compounds 2, 4, and 6

**Figure 13:**
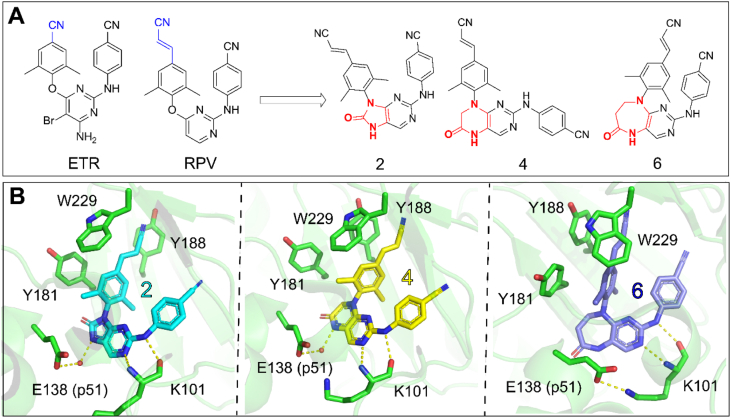
(A) Design of novel bicyclic DAPY NNRTIs **2**, **4**, and **6** from ETV and RPV; (B) Co-crystal structures of HIV-1 RT with **2** (PDB: 8FCC), **4** (PDB: 8FCD), and **6** (PDB: 8FCE).

Janeba's team has done outstanding work on the tolerant region II in 2023. They substituted the pyrimidine core of ETV and RPV with modified bicyclic cores to introduce additional polar moiety, which led to the compounds **2**, **4**, and **6** (Fig. 13A)^49^. The compounds carrying acrylonitrile moiety displayed single-digit nanomolar activities against the wild-type (WT) virus (EC_50_ = 2.5, 2.7, and 3.0 nmol/L, respectively), where the low nanomolar activity was retained against HXB2 (EC_50_ = 2.2‒2.8 nmol/L) and the K103N and Y181C mutated strains (fold change, 1.2×‒6.7×).

As shown in Fig. 13B, all three compounds maintained the double hydrogen bonds between the aniline nitrogen and the K101 backbone carbony, as well as between the pyrimidine core nitrogen and the K101 backbone nitrogen. Due to an opening and solvent-accessible area (the tolerant region II) around the core, there is water observed interactaction with the core in the five- and six-membered structures of compounds **2** and **4**, but not with the seven-membered compound **6**. This water was coordinated by E138 from the p51 subunit, further leading to the forming of the water-mediated hydrogen bond between compounds and E138. In the binding of compound **6** to NNIBP, E138 was shown to form a salt bridge, which confers 2‒3-fold resistance in cell culture. More interestingly, the introduction of modified bicyclic cores gave an additional polar moiety, which resulted in increased aqueous solubility. Specifically, compound **2** exhibited significantly improved phosphate-buffered saline solubility (10.4 μmol/L) compared to ETV and RPV (<< 1 μmol/L). The charm of the modification targeted tolerant II is to improve the drug resistance profiles while enhancing the solubility of DAPY NNRTIs.

##### 2.2.1.3 JLJ604

**Figure 14:**
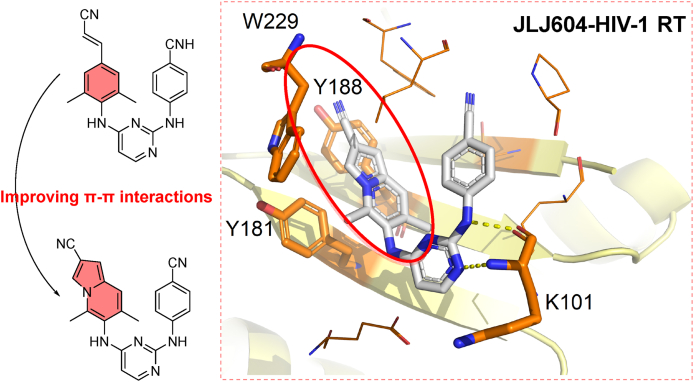
Crystal structure of HIV-1 RT in complex with **JLJ604** (PDB code: 7SNZ).

Based on the co-crystal structure of RPV/HIV-1 RT (Fig. 6B), Lee et al.^50^ replaced the cyanovinyl pheny in the hydrophobic channel with a CN-indolizine to enhance the *π‒π* interactions with Y181, Y188, and the highly conserved W299, yielding the potent HIV-1 NNRTIs **JLJ604**. The introduction of the CN-indolizine group led to excellent inhibitory activities against HIV-1 WT (EC_50_ = 1.0 nmol/L), the single mutant Y181C (EC_50_ = 0.57 nmol/L), and the double mutant K103N/Y181C (EC_50_ = 39.0 nmol/L) strains. Subsequently, the co-crystal structure of **JLJ604** in complex with RT was reported by Frey et al.^51^ in 2022. The binding mode of **JLJ604** is similar to that of RPV, enabling it to maintain high potency against RT mutants. As expected, the left-wing CN-indolizine group of **JLJ604** further penetrated into the hydrophobic tunnel and developed stronger *π‒π* interactions with Y188 and W229 (Fig. 14). **JLJ604** reduced the effect with Y181 and contributed to its highly active against the mutant strain Y181C. Moreover, reduced interactions with mutable amino acids and increased interactions with conserved amino acids provide a successful reference for the further structural modification.

#### 2.2.2 Diaryltriazines (DATAs)

Diaryltriazines (DATAs) were first reported by Donald et al.^52^ in 2001. Although the solubility of the DATAs is greatly improved compared with DAPYs, poor potency against NNRTIs-resistant strains greatly limits its development. In recent years, great efforts have been made to improve its drug resistance profiles. Herein, the reported co-crystal structures of DATAs in complex with HIV-1 RT since 2010 were summarized and analyzed, hoping to enlighten the subsequent structural modifications.

##### 2.2.2.1 JLJ513

**Figure 15:**
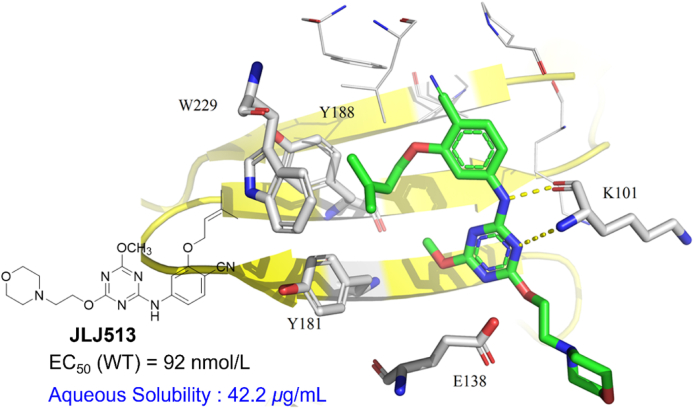
Crystal structure of HIV-1 WT RT in complex with **JLJ513** (PDB code: 4KKO).

Compound **JLJ513** was discovered by Bollini et al.^53^ in 2013 with EC_50_ of 92 nmol/L against WT HIV-1 and aqueous solubility of 42.2 μg/mL. As shown in Fig. 15, the classical double hydrogens with K101 are observed in the co-crystal structure of **JLJ513**/RT. However, unlike DAPY NNRTIs, the dimethylallyl group of benzene ring occupied the tunnel rather than the left-wing substituent of DAPYs, resulting in the sharply decreased *π‒π* interactions between **JLJ513** and the residues W229, Y181, and Y188, which was responsible for an approximately 100-fold reduced potency against WT RT than that of RPV. In addition, the introduction of water-soluble morpholine rings can explain the 350-fold increase in solubility over RPV.

##### 2.2.2.2 JLJ527, JLJ529, JLJ564, JLJ605 and JLJ639

**Figure 16:**
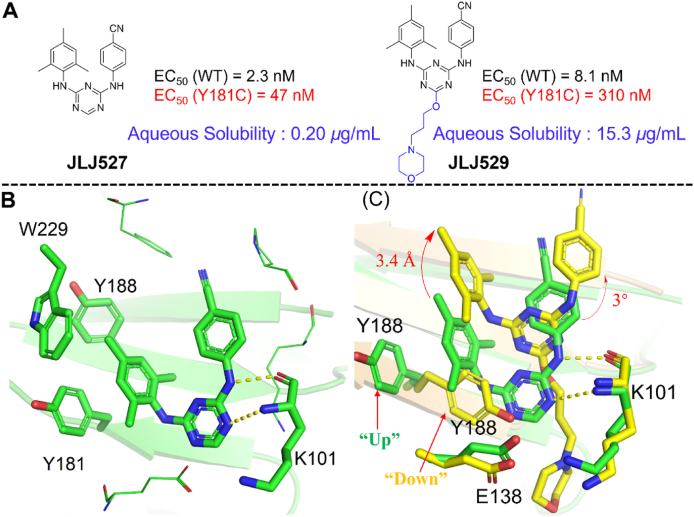
(A) Chemical structures of **JLJ527** and **JLJ529**. (B) Crystal structures of HIV-1 WT RT/**JLJ527** (PDB code: 4O4G) and (C) Superposition of HIV-1 RT/**JLJ529** (PDB code: 4O44) (yellow) onto HIV-1 WT RT/**JLJ527** (PDB code: 4O4G) (green).

To improve the activity of **JLJ513**, a series of novel DATAs were designed through computer-aided and structure-based drug design by Bollini et al^54^. Amon them, **JLJ527** and **JLJ529** were demonstrated with promising activity against HIV-1 WT and mutant strains (Fig. 16A).

**Figure 17:**
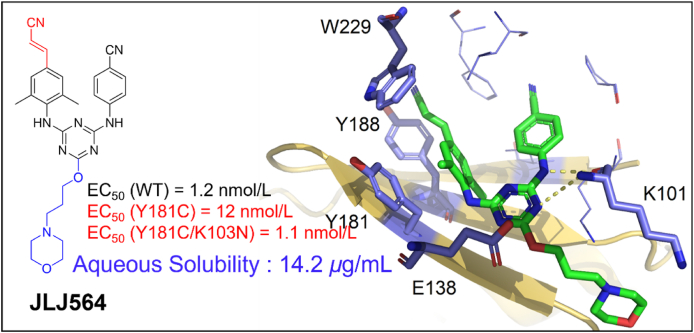
Crystal structure of HIV-1 WT RT/**JLJ564** (PDB code: 7SO1).

The co-crystal structure of **JLJ527** and **JLJ529** in complexes with HIV-1 RT was reported by Andrea et al.^54^ in 2014. Same as DAPYs, **JLJ527** adopts the classical horseshoe-like conformation in NNIBP and takes part in extensive van der Waals interactions with the residues K101, K103, Y181, Y188, P227, W229, and L234 of the p66 subunit (Fig. 16B). The left wing of **JLJ527** extends into the hydrophobic tunnel and form aryl–aryl interactions with the surrounding residues Y181, Y188 and W229, the double hydrogen bonds formed by the central triazine core and the right-wing secondary amine of **JLJ527** with the backbone of K101 were also observed. Compared with **JLJ527**, the novel morpholinopropoxy of **JLJ529** penetrated into the RT solvent interface through the entrance channel and caused the overall conformation of **JLJ529** to be shifted by 3.4 Å deeper and rotated by ∼3°, destroying the double hydrogen bonds between inhibitors and K101 (Fig. 16C). Furthermore, the residue Y181 adopted a “down” conformation different from the classical “up” conformation in the co-crystal structure of **JLJ529** in complex with HIV-RT, which led to a serious reduction in the activity against Y181C mutant (EC_50_ = 310 nmol/L). However, the morpholine ring successfully increased the solubility of JLJ529 (*S* = 15.3 μg/mL) compared to **JLJ527** (*S* = 0.20 μg/mL). Further structural modification was conducted by Frey et al.^51^ based on the co-crystal structure of **JLJ529**/RT, with the aim to increase the activity and solubility simultaneously. Replacement the methyl group on the benzene ring of **JLJ564** with a cyano vinyl group yielded **JLJ564**, which exhibited EC_50_ values of 12 and 1.1 nmol/L against the mutant Y181C and K103N/Y181C, being about 21‒28 times potent than that of **JLJ529**. As expected, **JLJ564** also has a good solubility *(S* = 14.2 μg/mL). As shown in Fig. 17, the introduction of cyano vinyl group makes **JLJ564** move towards the upper of NNIBP compared to that of **JLJ529**, thus maintaining the double hydrogen bond with the backbone of K101. Importantly, the cyano vinyl group goes deep into the hydrophobic channel and enhances the aryl–aryl interactions with the conserved W229, which contributes to its improved activity.

**Figure 18:**
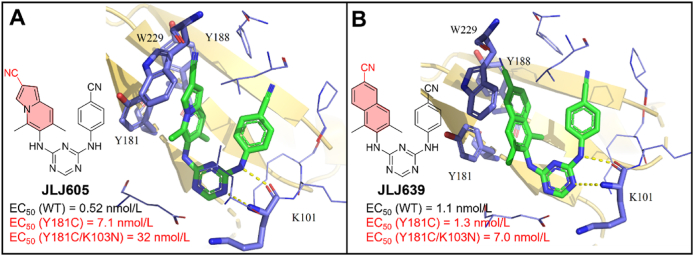
(A) Crystal structures of HIV-1 WT RT/**JLJ605** (PDB code: 5C24) and (B) HIV-1 WT RT/**JLJ639** (PDB code: 5C25).

Meanwhile, a series of novel DATAs NNRTIs were designed based on the co-crystal structure of **JLJ527/**RT (Fig. 16B) by Lee et al.^50^ in 2015, **JLJ605** and **JLJ639** were identified with significantly improved potency against HIV-1 WT, Y181C and K103N/Y181C mutants (Fig. 18A and B).

The co-crystal structures demonstrated that the introduction of 7-indolazinylamino and naphthylamine group of **JLJ605** and **JLJ639** enhanced the *π‒π* interactions with the hydrophobic channel, thus improving their potency to Y181C and K103N/Y181C. Especially, **JLJ639** displayed a 36- and 13-fold increase in potency against Y181C and K103N/Y181C than that of **JLJ527**, respectively. The work further demonstrated that the stronger interactions with conserved amino acids can improve the compounds resistance profiles.

#### 2.2.3 Catechol diethers

**Figure 19:**
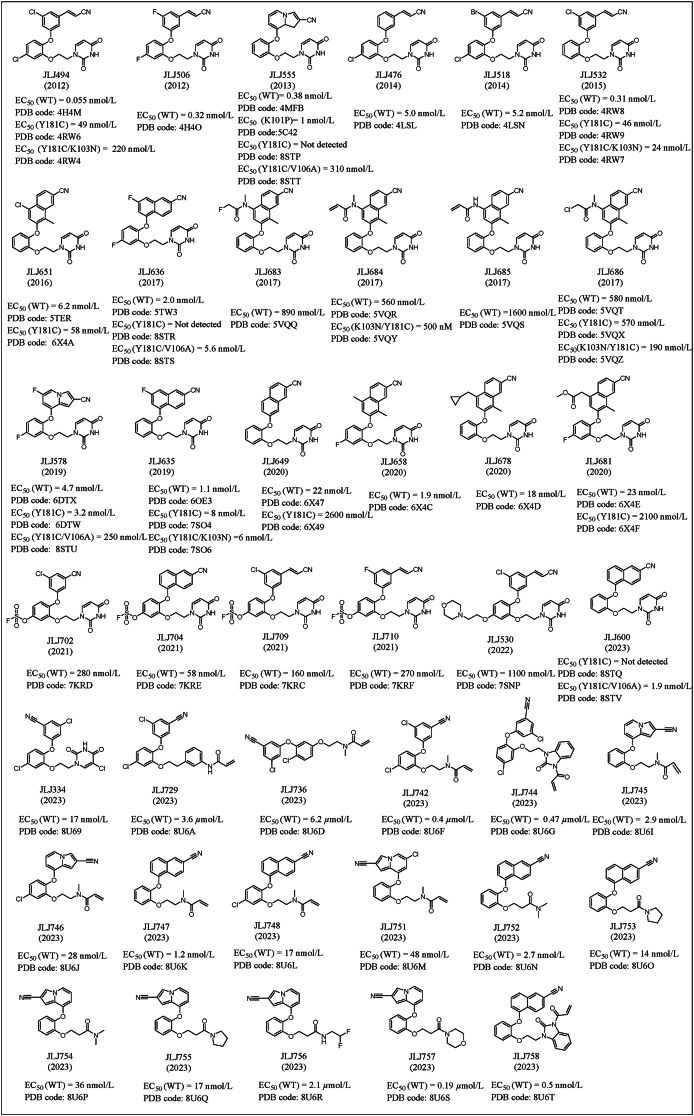
The chemical structures of 41 catechol diethers with co-crystal structures since 2012.

In 2001, Mariela et al.^55^ first identified catechol diethers as novel NNRTIs through computer-aided drug design. A series of catechol diethers with novel structures and mechanisms have been developed after 20 years of development. Here, the co-crystal structures of catechol diethers/RT reported in the last 12 years were summarized to elucidate the implications of structural biology for drug design (Fig. 19)^56^, ^57^, ^58^, ^59^, ^60^, ^61^, ^62^, ^63^, ^64^, ^65^, ^66^, ^67^.

##### 2.2.3.1 JLJ494, JLJ532, JLJ578, and JLJ635 JLJ494

**Figure 20:**
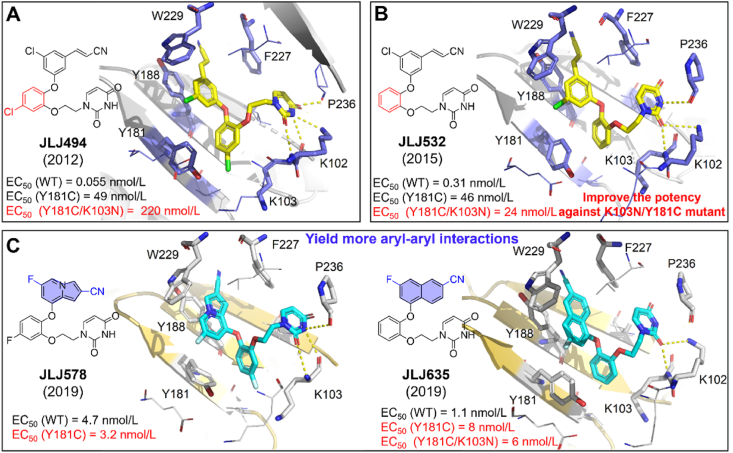
(A) Crystal structure of HIV-1 RT/**JLJ494** (PDB code: 4H4M), (B) HIV-1 RT/**JLJ532** (PDB code: 4RW8) and (C) HIV-1 RT/**JLJ635** (PDB code: 6DTX) and HIV-1RT/**JLJ578** (PDB code: 6OE3).

The compounds were reported by Mariela et al.^55^ in 2011, and it is still one of the best compounds against HIV-1 WT strain, exhibiting an EC_50_ value of 0.055 nmol/L (Fig. 20A). A series of modifications to **JLJ494** led to the discovery of JLJ532, JLJ578, and JLJ635 (Fig. 20B and C)^63^^,^^64^.

Kathleen et al.^57^ identified the co-crystal structure of **JLJ494/**RT in 2012. As depicted in Fig. 20A, the halocyanovinylphenyl (HCVP) group of **JLJ494** reached into the “tunnel” composed of Y188, W229, and F227, forming the *π‒π* interactions with Y188 and W229. The residue Y181 takes an uncommon “down” conformation, which differs from its “up” conformation in the co-crystal structure of DAPYs/RT, resulting in the disappearance of the *π‒π* interactions between **JLJ494** and Y181. Notably, HCVP could form stronger van der Waals contacts with the conserved residues W229 and F227 to compensate for the missing interaction with Y181. Meanwhile, the catechol ring is close to residues V106 and Y181, and the chlorine atom on C5 protrudes to the “entrance” near K103. The ethoxy linker interacts with V106 and F227 to locate the terminal uracil ring in the “groove”. The uracil group formed four hydrogen bonds with K10, K103, and P236, which accounted for the excellent potency against HIV-1 WT strain. However, the activity of **JLJ494** against the double mutant K103N/Y181C sharply decreased, with EC_50_ values of 220 nmol/L. With **JLJ494** as lead, multiple rounds of modifications were carried out by Kathleen et al.^59^, and **JLJ532** was identified with significantly improved potency against K103N/Y181C. Then, the co-crystal structures of HIV-1 WT RT, Y181C RT, and K103N/Y181C RT in complex with **JLJ532** have also been determined to clarify its increased resistance profiles.

**Figure 21:**
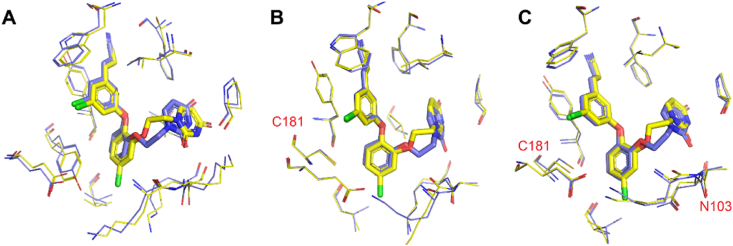
Superposition of **JLJ494**/RT and **JLJ532**/RT. (A) WT RT in complex with **JLJ494** (yellow) (PDB code: 4H4M) aligned with WT RT in complex with **JLJ532** (blue) (PDB code: 4RW8). (B) Y181C RT in complex with **JLJ494** (yellow) (PDB code: 4RW6) aligned with Y181C RT in complex with **JLJ532** (blue) (PDB code: 4RW9). (C) K103N/Y181C RT in complex with **JLJ494** (yellow) (PDB code: 4RW4) aligned with K103N/Y181C in complex with **JLJ532** (blue) (PDB code: 4RW7).

**Figure 22:**
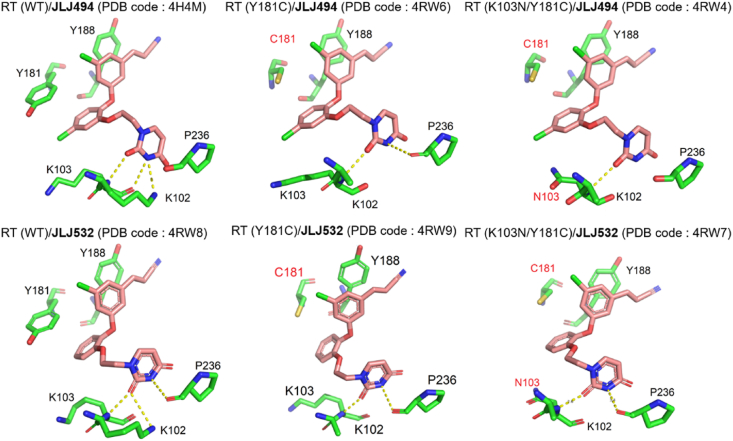
Ethoxy uracil conformation of compounds **JLJ494** and **JLJ532** in the RT (WT), RT (Y181C), and RT (K103N/Y181C).

Compared to **JLJ496**, **JLJ532** removed the chlorine atom on the central catechol ring, is approximately 6-fold less susceptible to WT RT, and has comparable potency to Y181C (Fig. 20B). However, the potency against K103N/Y181C was increased by 10-fold^59^. The results could be explained by two unique conformations of the ethoxy uracil side chain: *syn-anti-gauche* (*sag*) and *anti-anti-gauche* (*aag*) conformations. The different orientation in the ethoxy linker can be clearly observed in the superposition of **JLJ494**/RT (*sag* orientation) and **JLJ532**/RT (*aag* orientation) (Fig. 21). Although the *sag* conformation of **JLJ494** is the dominant conformation for WT RT, it is unfavorable for Y181C and K103N/Y181C RT, resulting in a rapid decline in potency against K103N/Y181C. It has been reported that the 5-Cl in the catechol ring of **JLJ494** is involved in the rotation of ethoxy uracil. Due to the presence of 5-Cl in the catechol ring, a gap is formed in the “entrance” channel of K103N/Y181C RT, which rotates the ethoxy uracil of **JLJ494** and causes two hydrogen bonds of **JLJ494**/RT lost (Fig. 22). However, the ethoxy uracil of **JLJ532** maintains the *aag* orientation in WT RT, Y181C RT and K103N/Y181C RT, which allows it to maintain two hydrogen bonds in K103N/Y181C RT and has favorable potency against mutant strains.

Based on the co-crystal structure of HIV1-RT/**JLJ532** (Fig. 20B), **JLJ578** and **JLJ635** were obtained with improved activity against Y181C and K103N/Y181C (EC_50_ = 3.2‒8 nmol/L) by Sasaki T and Kudalkar SN in 2019^63^^,^^64^. **JLJ578** and **JLJ635** replace the HCVP group with 4-cyano indolizine and naphthalene to increase the hydrophobic interactions with the conserved residue W299, respectively. Both inhibitors increased their potency 6-15-fold against Y181C and K103N/Y181C mutants. In contrast to **JLJ532**, the uracil group of **JLJ578** and **JLJ635** develops new hydrogen bonds with the backbone of K103 and P236 as hydrogen bond donors and acceptors, respectively (Fig. 20C), which makes their increased potency.

##### 2.2.3.2 JLJ702, JLJ709, and JLJ710

Covalent inhibitors are a rapidly growing discipline in drug discovery. Based on the co-crystal structure of **JLJ494**/HIV-1 RT (Fig. 20A), the fluorosulfate warhead was introduced to the catechol diethers NNRTIs yielding three Y181-target covalent inhibitors **JLJ702**, **JLJ709** and **JLJ710**^65^.

**Figure 23:**
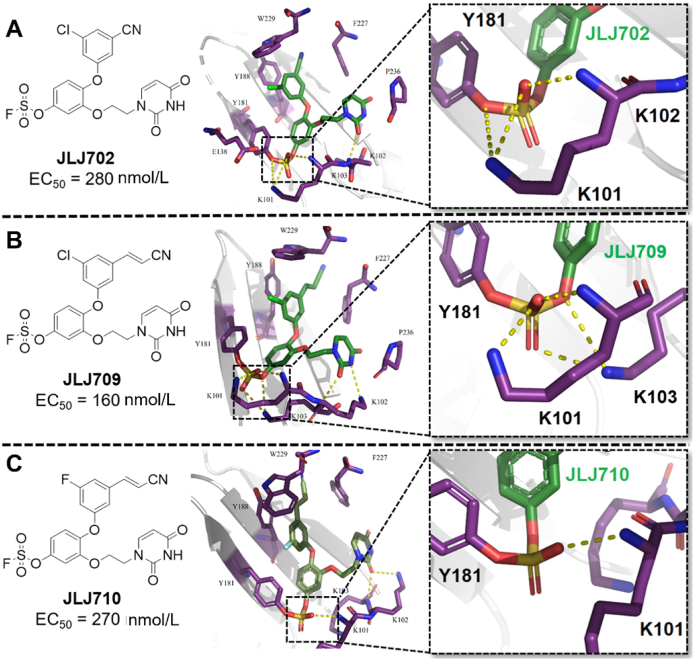
(A) Crystal structure of HIV-1 RT/**JLJ702** (PDB code: 7KRD). (B) Crystal structure of HIV-1 RT/**JLJ709** (PDB code: 7KRC). (C) Crystal structure of RT/**JLJ710** (PDB code: 7KRF).

As shown in Fig. 23, the structure demonstrated that the fluorosulfate warheads of the three compounds covalently bind to the hydroxy of Y181. However, covalent binding may hinder the “wiggling” and “jiggling” movement of compound in NNIBP, resulting in partial disappearance of the hydrogen bond network formed by uracil and NNIBP. Although the newly introduced fluorosulfate warheads formed new hydrogen bonds with K101, K102, and K103 to compensate for the missing, their activity sharply decreased (EC_50_ = 160‒280 nmol/L) compared to that of **JLJ494**. We attributed the failure to the fact that Y181 is the amino acid with a high frequency of mutations in NNIBP, and designing covalent inhibitors targeting the mutable amino does not improve the resistance of compounds. Actually, covalent inhibitors targeting conserved amino acids have been shown to be an effective strategy to improve resistance.

##### 2.2.3.3 JLJ744 and JLJ758

Anderson's team proposed a covalent strategy for targeting K102 in HIV-1 RT to overcome drug resistance in 2023^68^. Totally, 34 compounds were synthesized and 21 compounds were characterized structurally with WT HIV-1 RT. Two of these inhibitors demonstrated covalent inhibitions for targeting K102 as evidenced by protein crystallography. Unfortunately, their ability to avoid drug resistance has not yet been identified.

**Figure 24:**
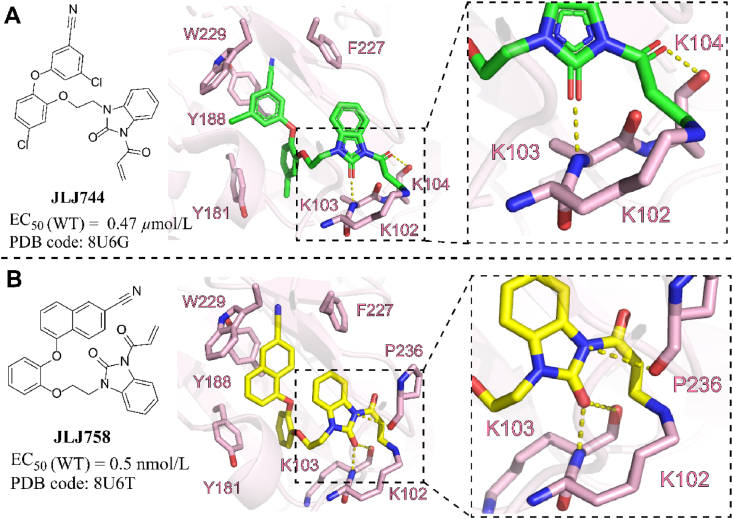
(A) Crystal structure of HIV-1 RT/**JLJ744** (PDB code: 8U6G). (B) Crystal structure of HIV-1 RT/**JLJ758** (PDB code: 8U6T).

**JLJ744** formed a 1.5 Å covalent bond with NZ of K102 through a Michael addition involving C_*β*_ of the acrylamide warhead (Fig. 24A). This structure also exhibited two hydrogen bonds at a length of 3.1 Å each to the backbone of the lysine quad. When compound **JLJ744** was redesigned with the 2-naphthonitrile head to increase potency, compound **JLJ758** also exhibited covalency in the crystallographic structure. Like the RT–**JLJ744** complex, this structure contained a 1.3 Å covalent bond involving NZ of K102 and C1 of **JLJ758** (Fig. 24B). Two noncovalent interactions are formed from **JLJ758** to the backbone of K103 at 2.8 and 3.3 Å, stabilizing the benzimidazolone linker. The warhead was further anchored by the linker through a hydrogen bond with the carbonyl of P236 at 3.5 Å.

##### 2.2.3.4 JLJ684 and JLJ686

**Figure 25:**
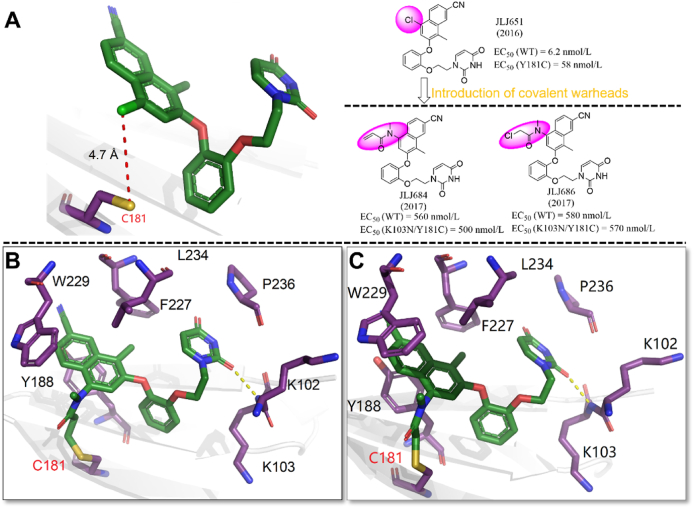
(A) Crystal structure of HIV-1 Y181C RT/**JLJ651** (PDB code: 6 × 4A). (B) Crystal structure of HIV-1 Y181C RT/**JLJ684** (PDB code: 5VQV). (C) Crystal structure of HIV-1 Y181C RT/**JLJ686** (PDB code: 5VQX).

As for mutable amino acids, covalent inhibitors targeting the mutated amino acids are another effective strategy. For the most common mutant strain Y181C in NNIBP, Chan et al.^62^ first reported two covalent NNRTIs (**JLJ684** and **JLJ686**) targeting the mutated C181 in 2017. Based on the co-crystal structure of **JLJ651**/Y181C RT (Fig. 25A)^66^, the acrylamide and chloromethylamide covalent warheads were introduced to the position of the chlorine atom of **JLJ651** naphthalene to design Y181C-target covalent inhibitors.

The co-crystal structures proved that **JLJ684** (Fig. 25B) and **JLJ686** (Fig. 25C) took a similar conformation like **JLJ651** to bind with Y181C HIV-1 RT. The naphthalene structure of both compounds can penetrate into the hydrophobic channel and form extensive hydrophobic interactions with W229, F227, and Y181, but multiple hydrogen bonds between urea and NNIBP disappeared. Although the sulfhydryl group of C181 can form covalent binding with the acrylamide warhead of **JLJ684** and the chloromethylamide warhead of **JLJ686** through nucleophilic substitution reaction, they all exhibited inferior activity compared to the lead **JLJ651**. Notably, **JLJ684** and **JLJ686** were demonstrated with comparable activity against WT and K103N/Y181C strains, proving the feasibility of designing covalent inhibitors targeting mutated amino acids^62^.

#### 2.2.4 Benzophenones

**Figure 26:**
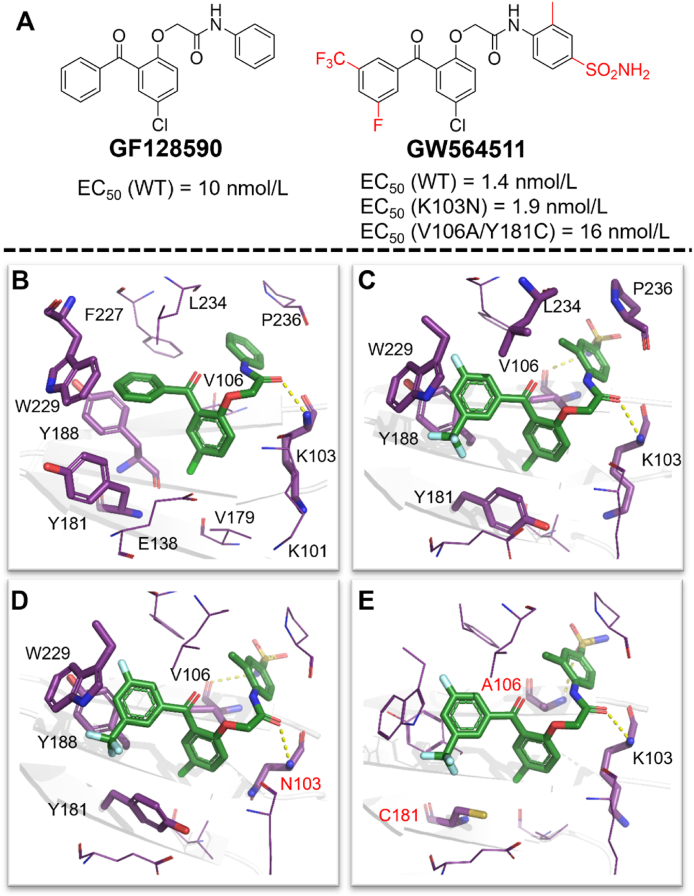
(A) The chemical structures of **GF128590** and **GW564511**. (B) Crystal structure of HIV-1 RT/**GF128590** (PDB code: 3DLE). (C) Crystal structure of HIV-1 RT/**GW564511** (PDB code: 3DLG). (D) Crystal structure of HIV-1 (K103N) RT/**GW564511** (PDB code: 3DM2). (E) Crystal structure of HIV-1 (V106A/Y181C) RT/**GW564511** (PDB code: 3DMJ).

Benzophenone analogues were first reported as HIV-1 NNRTIs in 1995. Among them, **GF128590** was structurally identified as an essential pharmacophore for antiviral activity with an EC_50_ value of 10 nmol/L against WT HIV-1 strains^69^. After that, Chan et al.^70^ synthesized compound **GW564511** with **GF128590** as lead in 2004. As expected, **GW564511** showed EC_50_ value of 1.4 nmol/L against WT HIV-1 strains and EC_50_ < 10 nmol/L against the common mutant HIV-1 strains (Fig. 26A).

**GF128590** has the left-wing benzene ring placed in the hydrophobic channel lined by the aromatic residues Y181, Y188, and the conserved W229, however, Y181 is easy to mutate, resulting in a loss of the *π‒π* interactions in the HIV-1 Y181C RT. The core benzene ring of **GF128590** can produce a large number of van der Waals interactions with the surrounding residues V106 and V179. Meanwhile, the right-wing benzene ring of **GF128590** is located in the groove above the NNIBP, close to the solvent (Fig. 26B). A hydrogen bond was observed between the carbonyl of amide group and the amino group of K103 main chain. This weak binding model makes **GF128590** intolerant to NNRTIs-resistant strains. Compared with **GF128590**, the introduction of meta-substituents onto left-wing benzene ring of **GF128590** made the left wing of **GW564511** deeper into the hydrophobic channel and formed a stronger van der Waals with the highly conserved W229, which leads to improved potency against the mutants^71^. Unexpectedly, the residue Y181 adopts the “down” conformation in NNIBP different from that of **GF128590**, which makes **GW564511** greatly reduce the *π‒π* interactions with Y181, thereby weakening the effect of Y181C mutation on the potency. Furthermore, the sulfonamide group of **GW564511** could extend to the solvent interface and form a hydrogen bond with V106 (Fig. 26C). In the co-crystal structures of K103N HIV-1 RT/**GW564511** (Fig. 26D) and V106A/Y181C HIV-1 RT/**GW564511** (Fig. 26E), new hydrogen bonds formed by the inhibitor with A106 or N103 could be observed, which compensated for the disappearance of the hydrogen bond due to the mutation. Interestingly, it was found that the bonding mode of **GW564511** in the mutant HIV-1 RT almost did not change, indicating that the benzophenones seemed to be more adaptable to the mutant NNRTI pocket than other NNRTIs.

In 2005, the benzophenone analogues **GW678248** with **GW564511** as the lead was reported. In biochemical assays, **GW678248** potently inhibited wild-type and mutant HIV-1 RT with EC_50_ between 0.8 and 6.8 nmol/L. In HeLa CD4 MAGI cell culture virus replication assays, **GW678248** had an EC_50_ < 21 nmol/L against a number of NNRTIs-resistant mutant strains, including L100I, K101E, K103N, V106A/I/M, V108I, E138K, Y181C, Y188 C/L, G190 A/E, P225H, and P236L and various combinations. Then, compound **GW695634**, an *N*-propionyl sulfonamide prodrug of **GW678248**, was designed to improve the solubility and bioavailability^72^^,^^73^. Unfortunately, neither **GW678248** nor its prodrug **GW695634** gave the co-crystal structures with WT HIV-1 RT. Interestingly, both compounds in complexes with single mutant RT (**GW678248** with K103N and **GW695634** with L100I) were obtained.

**Figure 27:**
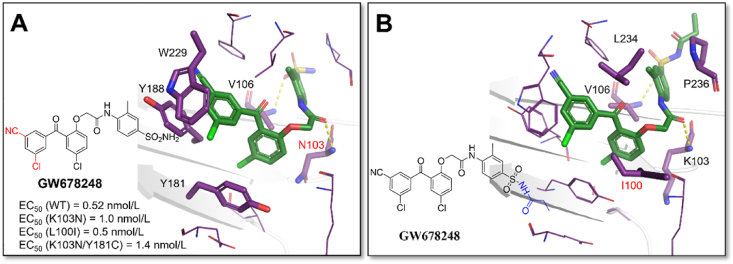
(A) Crystal structure of HIV-1 (K103N) RT/**GW678248** (PDB code: 3DOK). (B) Crystal structure of HIV-1 (L100I) RT/**GW695634** (PDB code: 3DOL).

As shown in Fig. 27, **GW678248** and **GW695634** adopted a similar conformation as **GW564511** in the HIV-1 K103N RT. The residue Y181 adopts the unexpected “down” conformation, which dramatically reduces the aromatic ring stacking contacts with **GW678248**. Additionally, on a cooperative level, the meta-substituent of both inhibitors pointed to the residues Y188 and the conserved W229, thus forming van der Waals contacts with these residues simultaneously. Further, there are additional van der Waals contacts between the central core of **GW678248** and the mutated side chain N103 (Fig. 27A). Compared with **GW678248**, the propionyl tail of its prodrug **GW695634** protruded to the RT solvent interface (Fig. 27B), which greatly increased the solubility and bioavailability.

#### 2.2.5 Indolyl aryl sulfones (IAS) and aryl-phospho-indole (APhI)

Indolyl aryl sulfones (IAS) is an attractive class of NNRTIs discovered by Williams et al.^74^ in 1993. In 2008, Zhao et al.^75^ designed and synthesized a series of indolyl sulfone derivatives and identified compound **7e** with excellent potency against wild-type HIV-1 RT (EC_50_ = 3.6 nmol/L).

**Figure 28:**
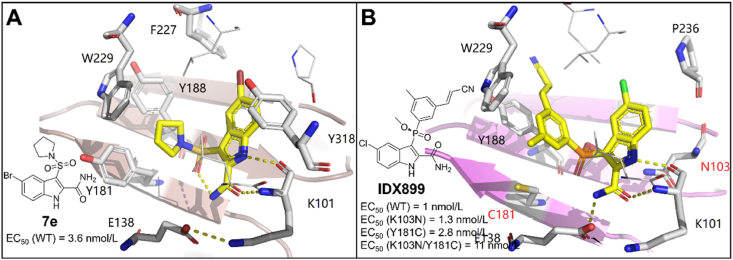
(A) Crystal structure of **7e** in complex with HIV-1 RT (PDB code: 2RF2). (B) Crystal structure of HIV-1 RT (K103N/Y181C) in complex with **IDX899** (PDB code: 5FDL).

In the co-crystal structure of HIV-1 RT/**7e**, the indole N and the amide carbonyl O can form the classical double hydrogen bonds with the carbonyl and the nitrogen backbone of K101, respectively (Fig. 28A). Meanwhile, an intramolecular hydrogen bond was observed between an O of the sulfonamide and the indole 2-carboxamide NH in **7e**. The sulfone group increased the acidity of indole NH and allowed the formation of the hydrogen bond with K101, which has been proven to be a significant pharmacophore of indole aryl sulfones. Meanwhile, the central indole ring can form hydrophobic interactions with the conservative Y318.

However, the lack of hydrophobic interactions between **7e** and aromatic residues in hydrophobic channel led to the loss of activity against Y181C and K103N/Y181C mutants. Based on the co-crystal structure of **7e** in complex with HIV-1 RT, a series of derivatives with the novel aryl-phospho-indole (APhI) scaffold were designed through coordinated medicinal chemistry principles involving bioisosterism principle and molecular hybridization, and led to an aryl phosphinateindole candidate **IDX899** with potent *in vitro* activity against WT (EC_50_ = 1.2 nmol/L), K103N (EC_50_ = 1.3 nmol/L), Y181C (EC_50_ = 2.8 nmol/L) and K103N/Y181C (EC_50_ = 11 nmol/L)^76^, ^77^, ^78^, ^79^. **IDX899** successfully entered phase IIb clinical trial but was stopped due to the side effects of inducing seizures^80^. In 2016, Dousson C reported the co-crystal structure of **IDX899** in complex with HIV-1 (K103N/Y181C) RT^79^.

The co-crystal structure of **IDX899** in complex with K103N/Y181C RT indicated that the introduction of the acrylonitrile-phenyl group reached into the hydrophobic tunnel composed of Y188 and W229, forming important *π‒π* interactions with Y188, the propenyl group can form van der Waals contacts with the highly conserved residue W229 (Fig. 28B). In addition, a hydrogen bond between the NH of the indole ring and the carbonyl O of the K101 main chain can be observed. And the O atom and amino group of the amide group formed hydrogen bonds with the amino group of the side chain of K101 and the carbonyl oxygen of E138, respectively. Meanwhile, the indole ring of **IDX899** formed the aryl–aryl interactions with P236 and lipophilic interactions with the V106 and Y318^79^. Compared with **7e**, the additional *π‒π* interactions between **IDX899** and the hydrophobic tunnel made it an excellent activity against NNRTIs-resistant strains.

#### 2.2.6 tert-Butyldimethylsilyl-spiroaminooxathioledioxide (TSAO)

The co-crystal structure of the compound **TSAO-T** in complex with wild-type RT was reported by Das et al.^81^ in 2011, which made TSAO recognized as NNRTIs with the following characteristics: (1) TSAO has an embedded thymidine-analog nucleoside moiety structure, but the mechanism of action is different from that of NRTIs^82^^,^^83^; (2) TSAO destabilizes p66/p51heterodimer, whereas NNRTIs increase its stability^84^; (3) TSAO molecules are much larger than other NNRTIs; and (4) E138K is the most common resistant strain of TSAO^85^. However, the study of TASO has not achieved the satisfactory results to date.

**Figure 29:**
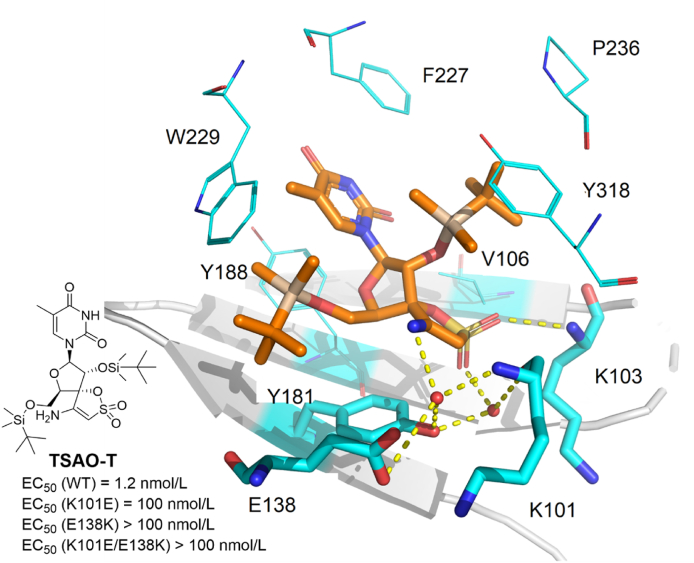
Crystal structure of HIV-1 RT in complex with **TSAO-T** (PDB code: 3QO9).

Although the inhibitory activity of **TSAO-T** against the NNRTIs-resistant strains is nearly lost, the co-crystal structure of HIV-1 RT**/TSAO-T** is helpful to understand its binding mode and mechanism^81^. Compared with other NNRTIs, the conformation of **TSAO-T** adopted a “dragon” shape in NNIBP, which enabled an unexpected expansion of the NNIBP and a rearrangement of Y181 and Y188 to accommodate its 5′-TBDMS group. Meanwhile, the 5′-TBDMS group interacted with E138 (p51) and W229, and there are van der Waals contacts between 2′-TBDMS and the residues P236, Y318, and F227 (Fig. 29). The thymine is located between Y188 and F227, stacking with F227. One of the sulfonyl oxygens on the oxthiane ring forms a hydrogen bond with the amino group of the K103 main chain, whereas, the other one forms a hydrogen-bond network mediated by two water molecules with E138, K101, and Y188, which leads to an excellent potency against WT HIV-1 strain. However, due to the strong molecular rigidity, it is difficult to adapt to the change of NNIBP in NNRTIs-resistant strains.

## 3 Novel allosteric sites of HIV-1 RT

**Figure 30:**
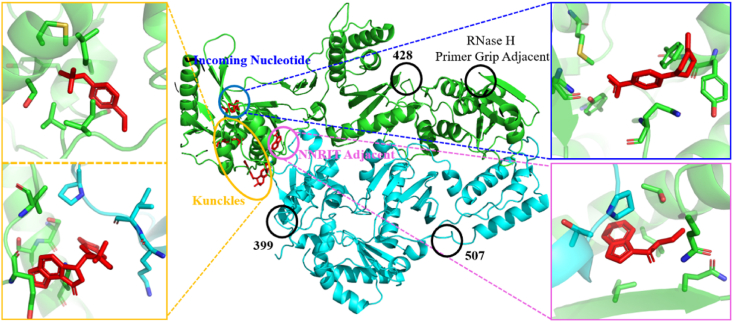
The fragment binding sites on HIV-1 RT (the first round).

Arnold'team detected HIV-1 RT by X-ray crystallographic fragment screening and identified seven allosteric sites, including the Incoming nucleotide binding, Knuckles, NNRTI adjacent, and 399 sites, located in the polymerase region of RT, and the 428, RNase H Primer Grip Adjacent, and 507 sites, located in the RNase H region (Fig. 30). And three sites (Knuckles, NNRTI adjacent and Incoming nucleotide binding) have shown inhibitory potency against HIV-1 RT, which have great development prospects^86^.

**Figure 31:**
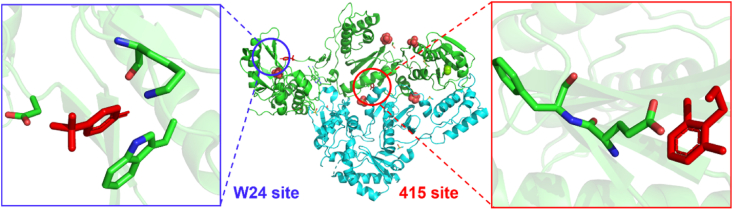
The fragment binding sites on HIV-1 RT (the second round).

Ten years later, this team developed a tool (Halo Library) for rapid identification of ligand binding sites on proteins using crystallographic fragment screening and conducted a new round of screening for HIV-1 RT^87^. Two sites not reported previously were found in this campaign (Fig. 31). The W24 site and 415 site, in addition to other potentially druggable sites to bind fragments in this campaign, are discovered.

Additionally, Losada et al.^88^ reported the crystal structures of three gp120 antagonists with HIV-1 RT in 2021, which were identified as bifunctional inhibitors capable of bridging NNRTI and NRTI binding sites.

### 3.1 NNRTI adjacent binding site: NNIAP

**Figure 32:**
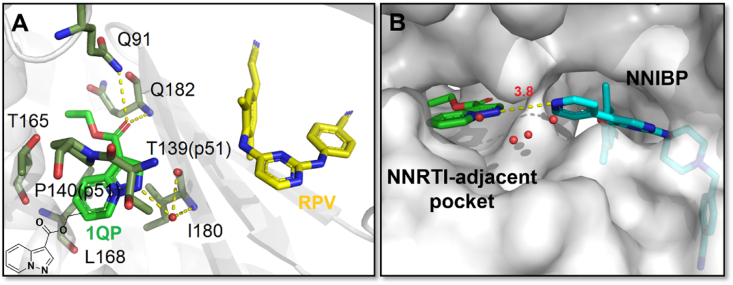
(A) HIV-1 reverse transcriptase with bound fragment 1QP at NNRTI adjacent site (PDB code: 4KFB). Select residues of the adjacent site of HIV-1 RT are shown as dark green sticks, with the fragment 1QP shown as bright green sticks. Hydrogen bonds are shown as yellow dotted lines. Water molecules are shown as red spheres. (B) Superposition of 16c/RT and 1QP/RT.

The NNRTI adjacent binding pocket (NNIAP), composed of the residues Q91, T165, L168, I180, Q182, T139 (p51), and P140 (p51), was found to be a tunnel-like pocket next to NNIBP and separated by the *β9* strand. A hydrogen-bond network formed by pyrazolo group with I180 through a water molecule was observed, which firmly located the fragment **1QP** in the pocket. Meanwhile, the carbonyl oxygen on the 3-carboxylate substituent could form two hydrogen bonds with the backbone amino group of the Q91 and the backbone amide nitrogen of the Q182, respectively. It is reported that the hydrogen bonds lead to a 2.1 Å C*α* backbone movement of Q91, affecting surrounding residues. Especially, a 1.1 Å C*α* backbone movement at Q182 results in a 1.5 Å C*α* movement of the catalytic tyrosine-methionine-aspartate-aspartate (YMDD) motif (Fig. 32A)^86^. The formation of the hydrogen bond network and the deformation of the YMDD motif are exactly the reasons for the HIV-1 inhibitory potency of fragment **1QP**.

**Figure 33:**
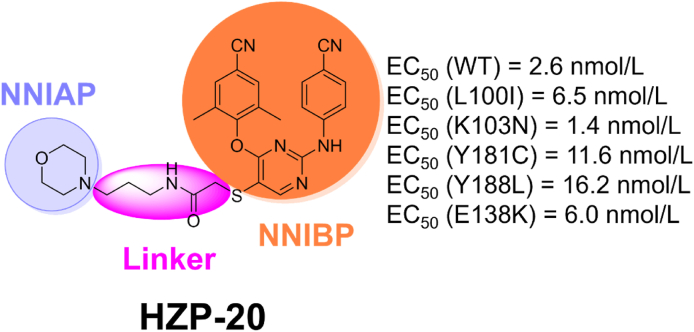
The chemical structure of compound **HZP-20**.

Significantly, a water molecule was observed 3.7 Å away from the RPV in NNIBP and 4.1 Å away from the fragment **1QP** in NNIAP, which makes it possible to design NNRTIs with a linker extending to the NNIAP composed of conserved residues. Meanwhile, the distance between pyridine ring of **16c** and fragment **1QP** is only 3.8 Å in the superposition of **16c**/RT and **1QP**/RT (Fig. 32B). The dual-site inhibitors targeting NNIBP and NNIAP may be a promising design idea to overcome the current mutant HIV-1 strains. Our laboratory designed and synthesized a series of DAPYs NNRTIs with high anti-HIV-1 potency in 2018 to simultaneously bind NNIBP and NNIAP^89^. Among them, compound **HZP-20** (Fig. 33) shows excellent activities against WT (2.6 nmol/L), L100I (6.5 nmol/L), K103N (1.4 nmol/L), Y181C (11.6 nmol/L), Y188L (16.2 nmol/L) and E138K (6.0 nmol/L) mutant strains. Unfortunately, compound **HZP-20** failed to get co-crystal structure in complex with HIV-1 RT.

### 3.2 Knuckles site

**Figure 34:**
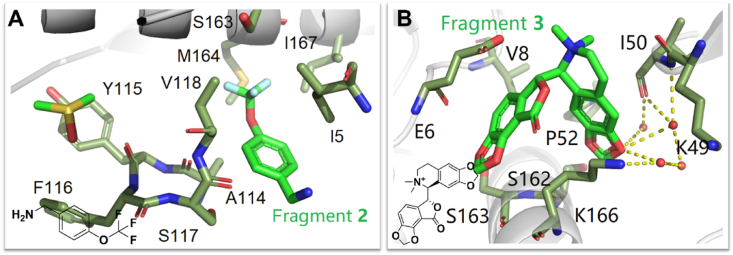
(A) HIV-1 RT with bound fragment 2 at the Knuckles site (PDB code: 4IFY). (B) HIV-1 RT with bound fragment 3 at the Knuckles site (PDB code: 4IG3).

The Knuckles pocket is a very promising allosteric inhibitory pocket in RT, which is a buried cavity near the polymerase active site prior to fragmentation. The binding of the fragments caused the surrounding residues to change and stabilize, forming the Knuckles pocket. As shown in Fig. 34A, the trifluoromethyl group of the fragment **2** can interact with M164 and S117 from a halogen bond. The aromatic ring of fragment **2** can form hydrophobic interactions with I5, A114, L214, and V118, but fragment **2** has no inhibitory potency against RT. After the fragment is bound to the pocket, residues S117 and I167 are exposed to the solvent, so that the space of the pocket is expanded in this direction, which leads to the discovery of fragment **3** (Fig. 34B). The fragment **3** can form hydrophobic interactions with K166, G51, and E6. Meanwhile, a hydrogen-bond network formed by fragment **3** with I50 and V8 can be observed in the co-crystal structure of **3**/HIV-1 RT, anchoring the fragment **3** in the pocket. Residues Y115 and F116, involved in polymerase activity, have a 3.2 Å shift in the backbone after pocket formation, which may explain the potency of the fragment **3** against HIV-1 RT.

### 3.3 Incoming nucleotide binding site

**Figure 35:**
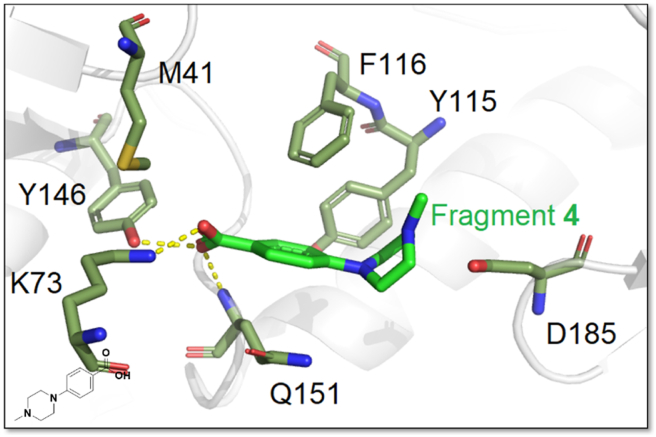
HIV-1 RT with bound fragment 4 at the Incoming Nucleotide Binding site (PDB code: 4ICL).

The promising Incoming nucleotide binding pocket, composed of Y115, F116, Q151, and D185, is located next to the polymerase active site. The fragment **4** has inhibitory potency against RT, with an IC_50_ value of 200 μmol/L. When fragment **4** binds to the pocket, the Q151 residue is repositioned, causing the pocket to expand to accommodate fragment **4**. Hydrogen bonds can be observed between the carboxylic acid group of **4** and the amino group of K73, the hydroxyl group of Y146 and the backbone amide of Q151, respectively. Meanwhile, the edge-to-face *π‒π* interactions can be seen, which are formed by the aromatic ring of **4** and Y115 and F116 (Fig. 35). Since D185 is a residue required for the polymerase active site, the electrostatic interactions between the piperazine of **4** and carboxylate of D185 are responsible for the potency of fragment **4** against HIV-1 RT. The inhibitor at the Incoming nucleotide binding site can directly interfere with dNTP binding, and the residues in this pocket are highly conserved, and thus HIV-1 is not susceptible to mutation, making this allosteric site a hotspot.

### 3.4 W24 site

**Figure 36:**
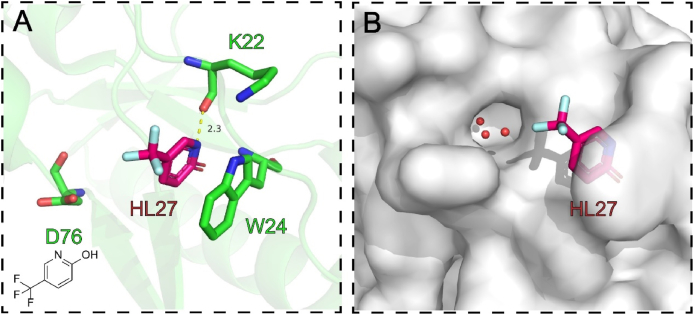
(A) The interactions between **HL27** and the residues of W24 site. (B) The solvent channel for fragment expansion (PDB code: 8DXG).

W24 lies in the finger subdomain of p66 and was found to be inhibitory for RT polymerase activity in our assay. **HL27** could form *π‒π* stacking with the indole ring of the well-conserved residue W24 (Fig. 36A)^87^. In addition, a short and presumably strong hydrogen bond (2.3 Å) was found between the aromatic ring nitrogen of the fragment and the main-chain carbonyl oxygen of K22. Importantly, a solvent channel close W24 site offered an opportunity to increase the volume of the pocket and to carry out the fragment-based drug design (Fig. 36B).

### 3.5 415 site

**Figure 37:**
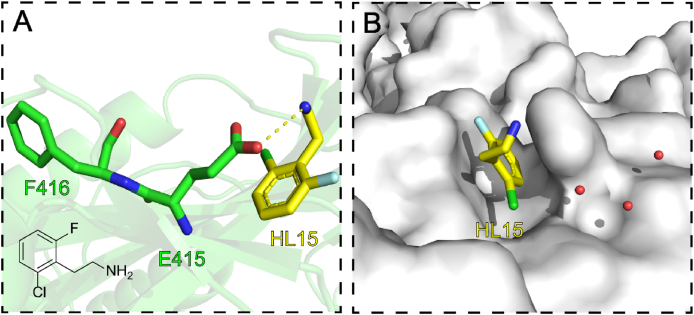
(A) The interactions between **HL15** and the residues of 415 site. (B) The solvent channel for fragment expansion (PDB code: 8DX8).

This newly identified site lies in the connection subdomain of p66. This pocket bound **HL15** by a hydrogen bond between the amino group of **HL15** and the side-chain oxygen of E415 (Fig. 37A). Ordered water molecules bridge interacted with F416 and provided extended channels for the fragment (Fig. 37B). **HL15** bound to inhibit RT activity at this site with an IC_50_ value of 2.1 mmol/L. Although their data were insufficient to suggest by which mechanism the RT was inhibited, there was a hypothesis to suggest that the process occurred allosterically^90^.

### 3.6 NBD binding pocket

In 2005, Debnath et al.^91^ discovered a new class of HIV-1 entry antagonists (NBD compounds), which can bind to F43 cavity of gp120 and prevent the HIV-1 virus from fusing with the host cell. In 2021, Lolada et al.^88^ determined co-crystal structures of three NBD compounds in complex with HIV-1 RT. Among them, compound NBD-14189 and NBD-14270 have shown potency anti-HIV-1 activity (EC_50_ < 200 nmol/L).

**Figure 38:**
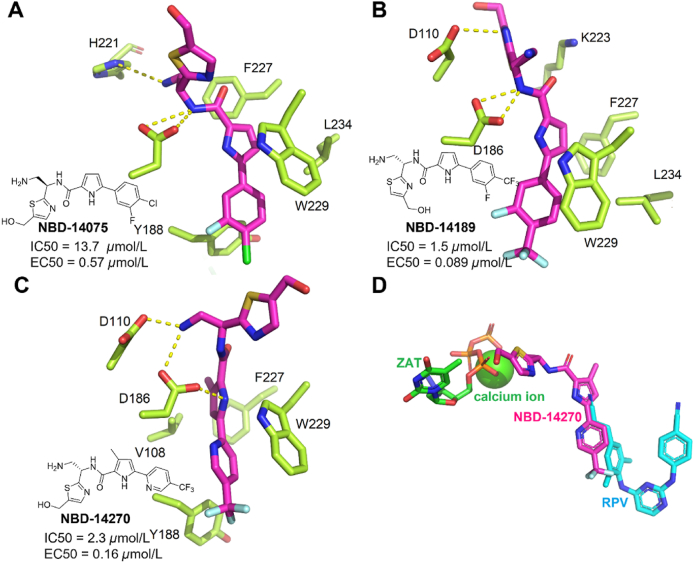
(A) Crystal structure of HIV-1 RT in complex with **NBD-14075** (PDB code: 7LQU). (B) Crystal structure of HIV-1 RT in complex with **NBD-14189** (PDB code: 7LPX). (C) Crystal structure of HIV-1 RT in complex with **NBD-14270** (PDB code: 7LPW). Select residues of the NNIBP of HIV-1 RT are shown as green sticks, with **NBD-14075**, **NBD-14270** and **NBD-14189** shown as red sticks. Hydrogen bonds are shown as yellow dotted lines. (D) Superposition of **NBD-14270/**RT, AZT-ZP/calcium ion/RT (PDB code: 5I42) and RPV/RT (PDB code: 2ZD1).

The NBD compounds bind to the region between NNIBP and NRTI binding site, located in the palm subregion of RT, which is called NBD binding pocket. As shown in Fig. 38A, the benzene ring of **NBD-14075** formed hydrophobic interactions with the conserved residue W229, which anchored the benzene ring in the hydrophobic channel composed of residue F227 and the highly conserved residue W229 in NNIBP. Meanwhile, the key hydrogen bounds can be observed between compound **NBD-14075** and the catalytic residue D186. Furthermore, **NBD-14075** formed a hydrogen bond with H221, the residue of NRTI site. And **NBD-14075** and **NBD-14189** could interact with the conserved F227 (Fig. 38B), while **NBD-14270** interacts with V108. Compared with **NBD-14075**, both **NBD14189** and **NBD-14270** can form an additional hydrogen bond with the catalytic residue D110, which is critical for the inhibition of HIV-1 RT (Fig. 38C). And the trifluoromethyl groups of the two compounds are further embedded in the hydrophobic channel, which adjusts the conformation of the benzene ring to interact with the highly conserved residue W229. The superposition of **NBD-14270/**RT, AZT-ZP/calcium ion/RT, and RPV/RT showed that the compounds can effectively bridge NNIBP and NRTI binding sites, which provides a great possibility for the design of dual-target inhibitors (Fig. 38D).

## 4 Concluding remarks and future perspectives

Since the first clinical diagnosis of AIDS in the 1980s, great efforts have been made in the field of AIDS treatment. However, the annual infection and death rate of AIDS is a constant reminder that AIDS is still a thorny infectious disease. Due to the lack of an AIDS vaccine, the development of anti-HIV drugs remains the standard treatment. HAART has significantly improved the quality of life of AIDS patients. NNRTIs are widely used in HAART regimens for their potent antiviral activity and high selectivity. Although there are six NNRTIs have approved by the FDA, a large number of NNRTIs-resistant strains have emerged due to the long-term use in clinical practice, which severely reduced their therapeutic effect. It underlines the demand to seek novel inhibitors with improved resistance profiles.

**Figure 39:**
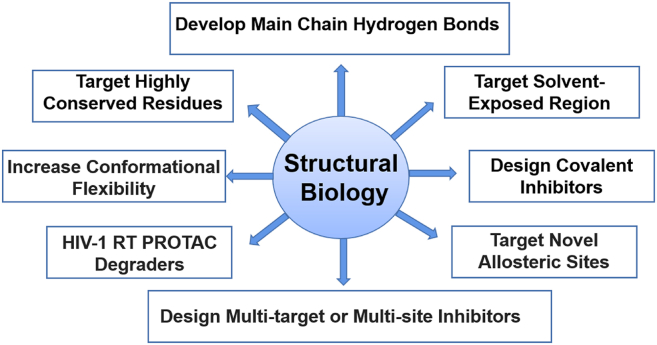
Future perspectives based on structural biology.

Structural biology has become an indispensable tool for deciphering drug targets, which can provide information on the structural biology of the targets, guide the structural optimization of the lead compounds, and give possibilities to combat emerging clinical resistance. As for allosteric drugs, structural biology can reveal the mechanism of action between allosteric drugs and targets, which transforms the discovery of allosteric drugs from traditional screening to precise drug design based on the co-crystal structures. Meanwhile, structural biology also provided an important basis for the discovery of novel RT allosteric sites, such as the Incoming nucleotide binding, Knuckles, and NNRTI adjacent site. In this section, we discussed the medicinal chemistry strategies targeting NNIBP and drug discovery based on novel allosteric sites and new mechanisms for combating drug resistance from the perspective of structural biology (Fig. 39).

### 4.1 Medicinal chemistry strategies

In the past decades, there has been a wealth of structural information on HIV-1 RT/NNRTIs complexes as well as a fundamental understanding of the mechanism of NNRTI-resistant mutations. Conformational flexibility, targeting highly conserved residues in NNIBP, developing main chain hydrogen bonds, and designing covalent NNRTIs were identified as important factors for combating the mutations.

Theoretically, if inhibitors can be adapted to the various binding pockets of mutant HIV-1 RT through conformational changes, then it is more likely to maintain the potency against the mutations. Actually, the second-generation DAPY NNRTIs have the ability of torsional flexibility, multiple conformations, repositioning, and reorientation, which can compensate to some extent for disturbance of resistance mutations and avoid the penalty of increasing binding energy^92^. On the other hand, inhibitors can adapt bioactive conformation, increasing affinity with HIV-1 RT and adaptability to a large number of NNRTIs-resistant mutations, which may improve the drug resistance profiles.

A widely accepted strategy, to reduce the effect of emergence and selection of NNRTIs mutations, would be to target the highly conserved residues in NNIBP to form strong interactions while decreasing the interactions with the easily mutant residues. There are four residues (F227, W229, L234, and Y318) in NNIBP that have been identified as highly conserved residues. Except for Y318, all the other residues are part of RT primer grip, and W229 is a prime residue candidate for targeted design of NNRTIs due to its physical participation in creating NNIBP^93^. Numerous RT/NNRTIs complexes studies suggested that the extension of the left wing of NNRTIs in the orientation of the highly conserved residue W229 could greatly increase the activity against the WT HIV-1 virus and its various mutations.

In order to prevent the reduction of binding affinity caused by mutations, extensive main chain hydrogen bonds or covalent binding between inhibitors and NNIBP can be developed. It was noted that the main-chain hydrogen bonds are unlikely to be disrupted by the NNRTI-resistant mutations and are significant for decreasing the free energy of binding, which acts as compensation for the decrease of the binding affinity. Besides, covalent inhibitors can form a stable covalent bond with the specific residues in NNIBP, which can completely knock out the mutation of the residues involved in covalent binding, and avoid the influence of the mutations of other residues on the affinity. In addition to the reported covalent modification strategies targeting Y181 and the mutant C181 residues, the important design concept targeting the conserved residues such as Y318 may offer a reliable strategy for combating drug resistance^62^^,^^65^.

Additionally, structure biology studies explored a broad space for modifications of NNIBP. For instance, RT/solvent interface of NNIBP was demonstrated to be an attractive site for introducing a novel pharmacophore to form multiple interactions or incorporating a moiety to improve the aqueous solubility and drug pharmacokinetics.

### 4.2 Drug discovery based on new allosteric sites and new mechanisms

The drug discovery acting at newly discovered binding allosteric sites or with novel mechanisms is urgently needed due to the rapid emergence of drug resistance to currently available antiretrovirals. With the development of structural biology, several original inhibitors focused on newly allosteric sites and with new mechanisms have been reported. Although their activity needs to be further improved, they have shown the potential as lead compounds for further modification.

Structural biologists have done a lot of innovative work in the discovery of novel druggable allosteric inhibitors on HIV-1 RT. Recently, Arnold et al.^86^ detected seven allosteric sites of HIV-1 RT by X-ray crystallographic fragment screening technology. Importantly, the ligands of the allosteric sites have shown inhibitory potency against HIV-1 RT, and drug design for these druggable sites is an attractive direction for researchers. Meanwhile, the discovery of these novel allosteric sites provides a possibility for the design of RT dual-site inhibitors. For example, the NNIAP ligand **1QP** with RT has a distance of 3.7 Å away from the NNRTIs and is connected by the tolerant region II, supporting the design of dual-site inhibitors targeting both allosteric sites^86^. Moreover, dual-site inhibitors also could be designed to target the polymerase active site and the allosteric site. Structural biology demonstrated that the polymerase active site was approximately 10 Å away from NNIBP, and the high specificity of NNRTIs can be used as an anchor to design dual-site inhibitors composed of a NRTI unit that joins to a NNRTI moiety through a linker^94^. Dual-site inhibitors can not only achieve complementary effects at each distinct target site but also combat the severe drug resistance caused by each single site.

Besides, dual-target inhibitors can improve the activity of inhibitors by regulating multiple steps of HIV-1 virus function and overcome the serious drug resistance of a single target, while avoiding limitations of drug combination therapies^95^. Currently, three HIV-1 entry antagonists (**NBD-14189**, **NBD-14075**, and **NBD-14270**) have been proven to be able to bind to HIV-1 RT and could be used designed as novel HIV-1 RT inhibitor^88^. The dual-target inhibitors can not only block the invasion process of HIV-1 but also inhibit the polymerization activity of HIV-1 RT, which can combat the drug resistance caused by a single mechanism.

Last but not least, proteolysis-targeting chimera (PROTAC) has been developed to be a powerful technology for drug discovery^96^^,^^97^. As for HIV-1 RT PROTAC degraders, structural biology can help to find the reasonable binding site of the protein of interest (POI) for the proteasome-mediated degradation of HIV-1 RT. More importantly, HIV-1 RT PROTAC degraders may be an appropriate approach to combat mutation-directed drug resistance of the small molecule-based, protein-interacting HIV-1 RT agents.

In summary, this perspective describes the structural biology advance of HIV-1 RT allosteric inhibitors and elaborates on the drug discovery based on the co-crystal structure of RT in complex with small molecules. Overall, the application of structural biology in drug design remains a continuing demand to achieve the transformation from traditional drug design to precise drug design based on crystal structure of target and ultimately obtain the novel RT inhibitors with potent activity, lower toxicity, and improved pharmacokinetic properties.

## Author contributions

Zhenzhen Zhou: Writing – review & editing, Writing – original draft. Yanying Sun: Writing – review & editing. Da Feng: Software. Zhao Wang: Supervision, Data curation. Fabao Zhao: Software. Shenghua Gao: Visualization. Peng Zhan: Writing – review & editing, Supervision, Resources, Funding acquisition. Dongwei Kang: Writing – review & editing, Supervision, Resources, Funding acquisition. Xinyong Liu: Writing – review & editing, Supervision, Resources, Funding acquisition.

## Conflicts of interest

The authors have no conflicts of interest to declare.
